# RB expression confers sensitivity to CDK4/6 inhibitor–mediated radiosensitization across breast cancer subtypes

**DOI:** 10.1172/jci.insight.154402

**Published:** 2022-02-08

**Authors:** Andrea M. Pesch, Nicole H. Hirsh, Anna R. Michmerhuizen, Kassidy M. Jungles, Kari Wilder-Romans, Benjamin C. Chandler, Meilan Liu, Lynn M. Lerner, Charles A. Nino, Connor Ward, Erin F. Cobain, Theodore S. Lawrence, Lori J. Pierce, James M. Rae, Corey W. Speers

**Affiliations:** 1 Department of Radiation Oncology,; 2 Department of Pharmacology,; 3 Program in Cellular and Molecular Biology, and; 4 Department of Internal Medicine, University of Michigan, Ann Arbor, Michigan, USA.

**Keywords:** Oncology, Breast cancer, DNA repair, Radiation therapy

## Abstract

Standard radiation therapy (RT) does not reliably provide locoregional control for women with multinode-positive breast cancer and triple-negative breast cancer (TNBC). We hypothesized that CDK4/6 inhibition (CDK4/6i) would increase the radiosensitivity not only of estrogen receptor–positive (ER^+^) cells, but also of TNBC that expresses retinoblastoma (RB) protein. We found that CDK4/6i radiosensitized RB WT TNBC (*n =* 4, radiation enhancement ratio [rER]: 1.49–2.22) but failed to radiosensitize RB-null TNBC (*n =* 3, rER: 0.84–1.00). RB expression predicted response to CDK4/6i + RT (*R*^^2^^ = 0.84), and radiosensitization was lost in ER^^+^^/TNBC cells (rER: 0.88–1.13) after *RB1* knockdown in isogenic and nonisogenic models. CDK4/6i suppressed homologous recombination (HR) in RB WT cells but not in RB-null cells or isogenic models of *RB1* loss; HR competency was rescued with RB reexpression. Radiosensitization was independent of nonhomologous end joining and the known effects of CDK4/6i on cell cycle arrest. Mechanistically, RB and RAD51 interact in vitro to promote HR repair. CDK4/6i produced RB-dependent radiosensitization in TNBC xenografts but not in isogenic *RB1*-null xenografts. Our data provide the preclinical rationale for a clinical trial expanding the use of CDK4/6i + RT to difficult-to-control RB-intact breast cancers (including TNBC) and nominate RB status as a predictive biomarker of therapeutic efficacy.

## Graphical abstract

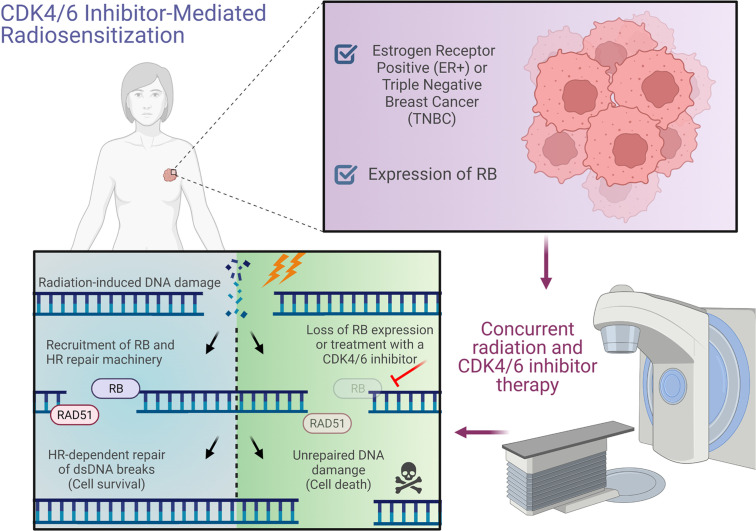

## Introduction

Breast cancer is a heterogeneous group of diseases in which treatment options — and often treatment outcomes — are tied to the presence or absence of molecular markers including the estrogen receptor (ER), progesterone receptor (PR), and the human epidermal growth factor receptor 2 (HER2). These receptors serve as drivers of tumorigenesis and disease progression, and ER^+^ or HER2-expressing tumors respond to inhibitors targeting either hormone-mediated or HER2-mediated signaling pathways, respectively. In triple-negative breast cancers (TNBC) that lack ER, PR, and HER2, this results in a limited number of targeted treatment options. Consequently, locoregional control remains a challenge in women with TNBC and multinode-positive ER^+^ breast cancers (1, 2).

Endocrine therapies including tamoxifen, fulvestrant, and aromatase inhibitors are used for the treatment of ER^+^ breast cancer following surgery, radiation therapy (RT), and/or chemotherapy (3). For patients whose cancers develop resistance to these therapies and who develop locoregional recurrences or metastatic disease, treatment with CDK4/6 inhibitors including palbociclib (4), ribociclib (5), and abemaciclib (6) has demonstrated an increase in progression-free survival time and decreased tumor metastasis (7). Mechanistically, pharmacological CDK4/6 inhibition (CDK4/6i) prevents phosphorylation of downstream cell cycle proteins such as RB that control cell cycle progression through the G_1_/S checkpoint (4). Although first approved in the metastatic setting for hormone receptor–positive (HR^+^) breast cancers, current clinical trials in HR^+^ breast cancer, including PALLAS (NCT02513394), monarchE (NCT03155997), PENELOPE-B (NCT01864746), and NATALEE (NCT03701334), seek to study the potential utility of CDK4/6 inhibitors in nonmetastatic HR^+^ breast cancer. Varying results have been reported, but based on improved invasive disease–free survival in the monarchE trial, abemaciclib was the first CDK4/6 inhibitor to be approved as an adjuvant therapy in women with high-risk, nonmetastatic, ER^+^ breast cancer with a high Ki67 score (8–10). Thus far, clinical trials using CDK4/6 inhibitors have been mostly restricted to women with ER^+^HER2^–^ breast cancer, and their potential role in treating TNBC remains unclear.

Multiple preclinical studies support the hypothesis that CDK4/6i can improve the efficacy of standard chemotherapy treatments (such as paclitaxel and cisplatin) in TNBC and other cancer types by inducing apoptosis or potentiating DNA damage and causing increased cell death (11–16). Previous studies combining low doses of CDK4/6i with RT demonstrate clinically meaningful radiosensitization in ER^+^ breast cancer models (17), though others have since suggested that combination treatment may be most effective when RT is administered prior to CDK4/6 inhibitor therapy (18). There is evidence to suggest that CDK4/6i can increase tumor cell sensitivity to proton therapy through impaired RAD51 foci formation (19), but at present, our understanding of the relationship between simultaneous radiation treatment and CDK4/6 inhibitor therapy in the treatment of TNBC is insufficient.

When compared with ER^+^ breast cancers, loss-of-function mutations in the retinoblastoma tumor suppressor *RB1* (RB) are more common in TNBC (7% versus <4%), and RB pathway alterations have been reported in up to 30% of TNBC (20, 21). *RB1* loss in breast cancer is associated with resistance to many therapies, including chemotherapy and radiation, and the presence of WT RB protein is an important determinant for the efficacy of CDK4/6 inhibitor monotherapy (4). However, in addition to CDK4/6-mediated phosphorylation of RB that is required to drive forward the G_1_/S cell cycle transition, there is growing evidence that RB is essential for multiple processes involved in the DNA damage response (22–24). For example, RB-null TNBC are more sensitive to γ-irradiation and less susceptible to CDK4/6 inhibitor monotherapy (4, 25, 26). Further studies also suggest that RB localizes to double-stranded DNA (dsDNA) breaks and helps recruit factors such as BRG1 necessary for proper dsDNA repair (27).

Taken together, the lack of targeted treatment options and the failure to achieve locoregional control of aggressive breast cancers — including TNBC — represents a clear unmet clinical need, but our understanding of the potential of combined CDK4/6 inhibitor therapy and radiotherapy in TNBC remains insufficient. In this study, we first sought to evaluate the efficacy of combining CDK4/6 inhibitors with radiotherapy in multiple preclinical models of TNBC. Next, we utilized isogenic models of breast cancer to explore how the presence or absence of RB led to changes in intrinsic cellular radiosensitivity and CDK4/6 inhibitor–mediated radiosensitization in ER^+^ and TNBC. Finally, we investigated the effects of RB expression on homologous recombination (HR) efficiency and the role of RB in CDK4/6 inhibitor–mediated radiosensitization both in vitro and in vivo.

## Results

### CDK4/6i radiosensitizes TNBC with WT RB1.

**Figure 1:**
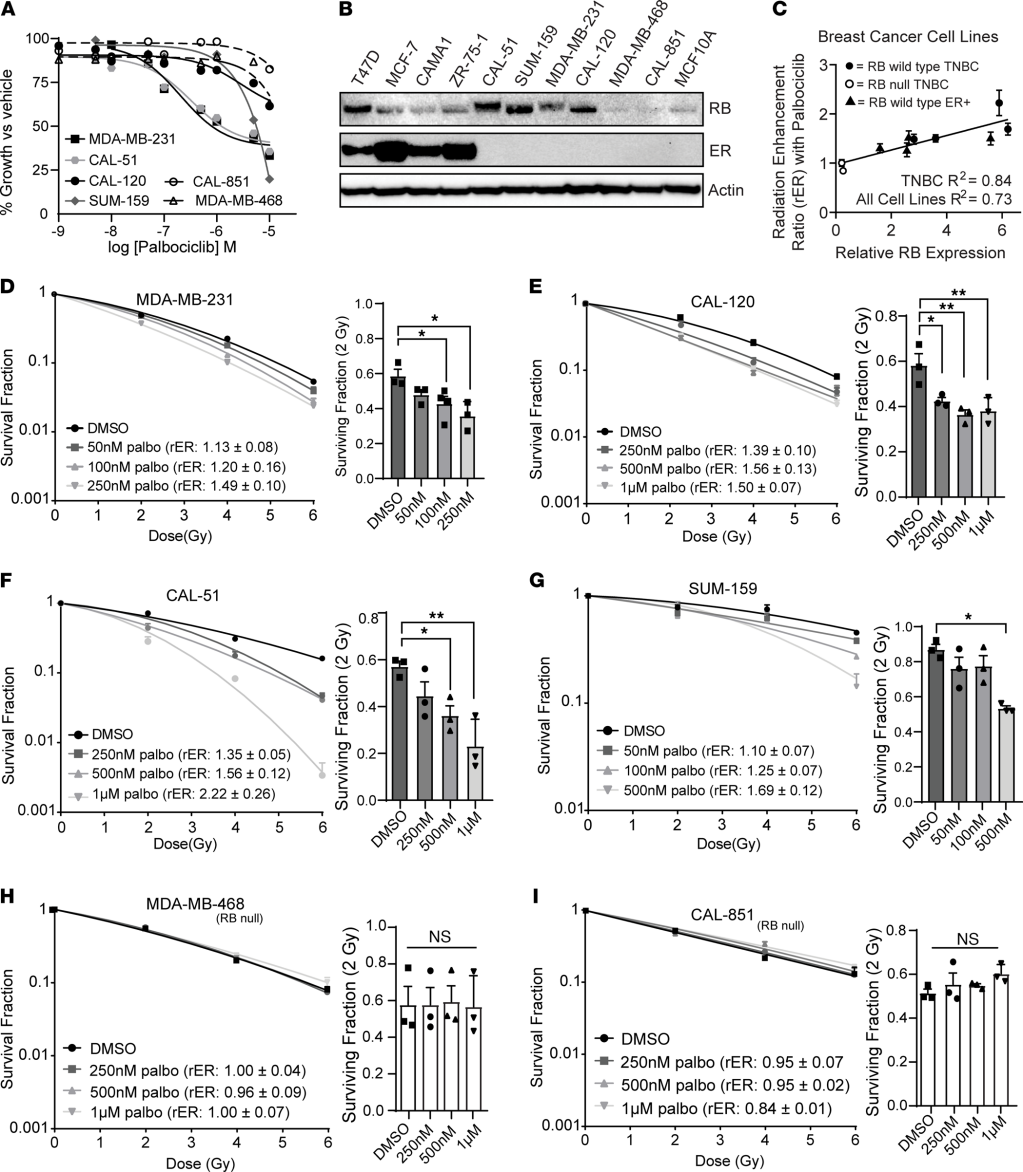
CDK4/6 inhibition with palbociclib radiosensitizes TNBC with WT *RB1*. (**A**) Cell viability (*n =* 3, graphed as average ± SEM) was measured in RB WT (solid lines) and RB-null (dashed lines) TNBC cell lines 72 hours after treatment with palbociclib. (**B**) Western blots were used to assess RB and estrogen receptor (ER) protein expression in various breast cancer cell line models. (**C**) RB expression was quantified using ImageJ and the correlation coefficient between RB expression and mean radiation enhancement ratios (rER) (highest concentration of palbociclib for each cell line) were compared between ER^+^ (solid triangles), WT *RB1* TNBC (solid circles), or RB-null TNBC (open circles). (**D**–**I**) Clonogenic survival assays were performed in the RB WT TNBC cell lines MDA-MB-231 (**D**), CAL-120 (**E**), CAl-51 (**F**), and SUM-159 (**G**), as well as the RB-null TNBC cell lines MDA-MB-468 (**H)**, and CAL-851 (**I**) cells with a 1-hour palbociclib pretreatment. Survival fraction of cells was calculated for each cell line at 2 Gy as the mean of 3 independent experiments and mean rER from 3 independent experiments are shown. A 1-way ANOVA with Dunnett’s post hoc test was used to compare palbociclib-pretreated groups to the vehicle-pretreated group. *P < 0.05; **P < 0.01.

To understand the single-agent effects of CDK4/6i on the proliferation of TNBC cell lines, we calculated the IC_50_ of proliferation after a 72-hour treatment with palbociclib, ribociclib, or abemaciclib (Figure 1A and Supplemental Figure 1, A and B; supplemental material available online with this article; https://doi.org/10.1172/jci.insight.154402DS1). All 3 drugs had minimal to moderate effects as monotherapies, especially in TNBC cell lines that lack expression of RB (MDA-MB-468 and CAL-851, IC_50_ > 1 μM), consistent with prior literature (4). RB expression varied across both ER^+^ and TNBC cell lines, and normalized breast epithelial tissue (MCF10A) expressed very low levels of RB (Figure 1B).

To determine if short-term CDK4/6i altered sensitivity of TNBC cell lines to ionizing radiation, we performed clonogenic cell survival assays using a 1-hour CDK4/6 inhibitor pretreatment with concentrations at or near the IC_50_ value for each TNBC cell line (Supplemental Tables 1 and 2). After a 1-hour pretreatment, the concentrations of all 3 CDK4/6 inhibitors used in both ER^+^ and TNBC cell lines led to a decrease in RB phosphorylation at both serine 780 (S780) and S807, suggesting that these concentrations and this time scale were sufficient to produce relevant physiological effects (Supplemental Figure 2).

A concentration-dependent increase in radiosensitization and a concentration-dependent decrease in cell survival was observed in TNBC cell lines with WT RB protein including MDA-MB-231 (radiation enhancement ratio [rER]: 1.49 ± 0.10), CAL-120 (rER: 1.50 ± 0.07), CAL-51 (rER: 2.22 ± 0.26), and SUM-159 (rER: 1.69 ± 0.12) with a 1-hour pretreatment of palbociclib before radiation (Figure 1, D–G). Pretreatment with ribociclib or abemaciclib led to similar levels of radiosensitization in MDA-MB-231 and CAL-120 cells (rER: 1.33–1.34 for ribociclib and 2.14–2.20 for abemaciclib; Supplemental Figure 1, D–G), demonstrating that all 3 drugs affected radiosensitization to a similar degree in vitro. To assess the time dependence of the combination therapy, we repeated clonogenic survival assays in MDA-MB-231 cells with sequential treatment (radiation treatment first followed by drug treatment 6 hours after RT; Supplemental Figure 1I). In this model, palbociclib-mediated radiosensitization was maintained at similar or slightly diminished levels compared with clonogenics performed with drug pretreatment prior to RT.

The observed rER values after CDK4/6i + RT suggest that this radiosensitization is similar in magnitude to that of other clinically used radiosensitizers such as cisplatin (28), with an rER threshold > 1.2. In contrast, in the TNBC cell lines MDA-MB-468 (rER: 0.96–1.0), CAL-851 (rER: 0.84–0.95), and BT-549 (rER: 0.91–0.93) that harbor inactivating *RB1* mutations, combination treatment with RT and palbociclib did not lead to radiosensitization (Figure 1, H and I, and Supplemental Figure 1H). In addition, combined palbociclib and RT in normal mammary epithelial (MCF10A) cells is not significantly more toxic than palbociclib treatment alone (Supplemental Figure 1C), suggesting that the combination treatment does not potentiate toxicities in normal breast tissue. Using rER values calculated from clonogenic survival assays (Figure 1, D–I) and the relative RB expression from each cell line (Figure 1B), we calculated the correlation coefficient between RB expression and the degree of palbociclib-mediated radiosensitization observed in vitro. RB mutant cell lines were not sensitized (average rER: 0.92 ± 0.05) to radiation after palbociclib treatment, whereas RB WT TNBC cells were radiosensitized (average rER at highest palbociclib concentrations: 1.66 ± 0.14; Figure 1C) by palbociclib. Enhancement ratios (Supplemental Table 3) for RB WT ER^+^ cell lines (17) were also predictive of efficacy of the combination therapy. Thus, RB expression status was predictive of CDK4/6 inhibitor–mediated radiosensitization in our models (*R*^2^ = 0.73 for all cell lines and *R*^2^ = 0.84 for TNBC only).

### CDK4/6i suppresses HR in TNBC.

**Figure 2:**
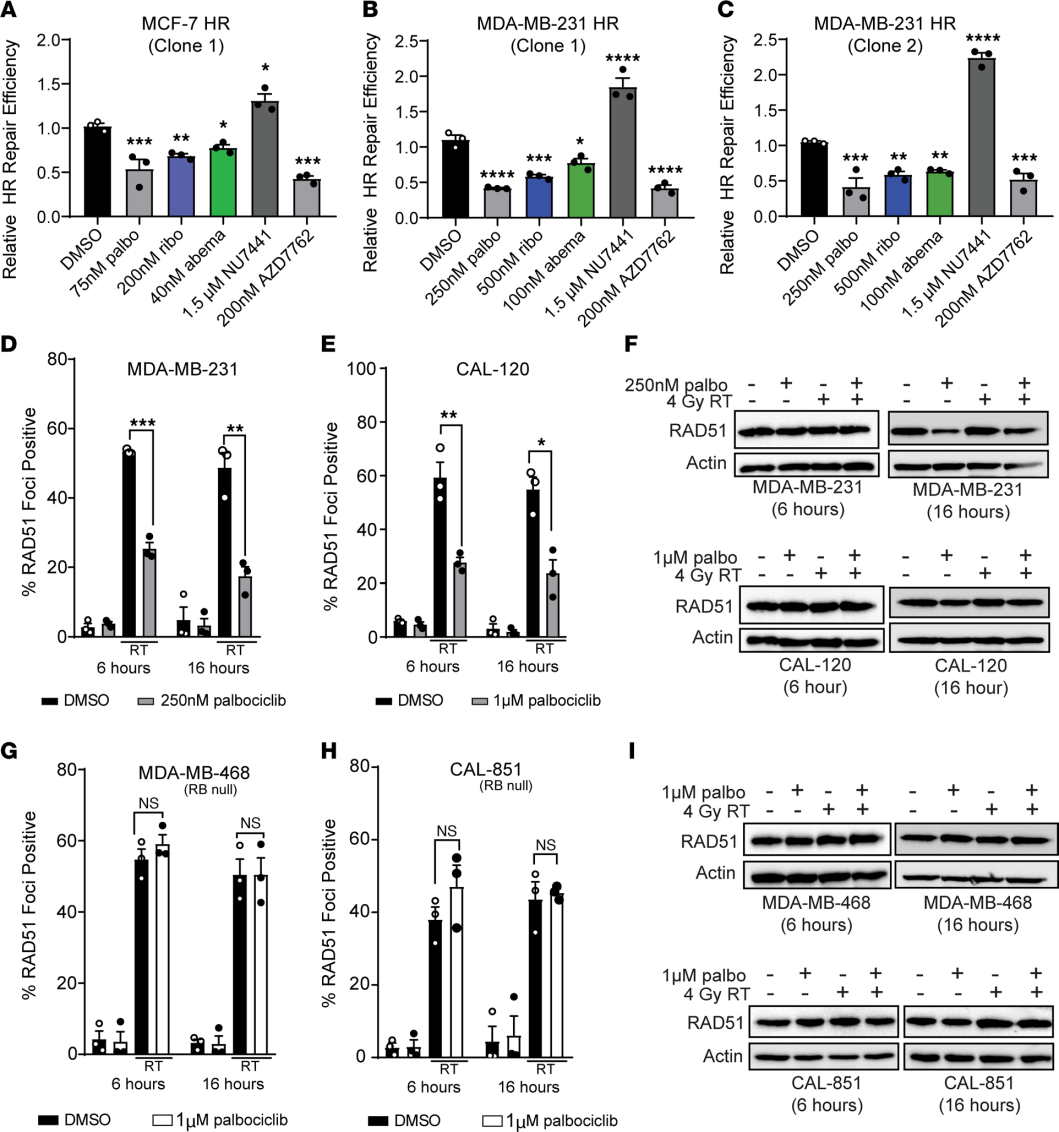
CDK4/6 inhibition suppresses HR in RB WT TNBC. (**A**–**C**) A GFP reporter system was used to measure relative HR repair efficiency in RB WT MCF-7 (**A**) and MDA-MB-231 (**B** and **C**) cells after treatment with in IC_50_ concentration of palbociclib (gray), ribociclib (blue), or abemaciclib (green). A CHK1/2 inhibitor (200 nM AZD7762) was used as a positive control, and a DNAPK inhibitor (1.5 μM NU7441) was used as the negative control. For the reporter assay a 1-way ANOVA with Dunnett’s post hoc test was used to compare treatment groups to DMSO-treated cells. *P < 0.05; **P < 0.01; ***P < 0.001, ****P < 0.0001. (**D**, **E**, **G**, and **H**) For RAD51 immunofluorescence, cells were pretreated for 1 hour with palbociclib, and coverslips were fixed 6 hours and 16 hours after 4 Gy radiation in RB WT MDA-MB-231 (**D**) and CAL-120 (**E**) cells, as well as RB-null MDA-MB-468 (**G**) and CAL-851 (**H**) TNBC cells. Two-tailed *t* tests were performed between radiation and combination treated groups at each RAD51 time point, and correction was performed for multiple comparisons. (**F** and **I**) Western blots were used to assess RAD51 protein expression at the same time points. All experiments represent the average of 3 independent experiments and bar graphs display the average ± SEM.

CDK4/6i is known to affect HR activity in ER^+^ breast cancer cells (17), which we confirmed in our experimental model system. We used a stable MCF-7 cell line expressing a GFP-based HR reporter system (29) and demonstrated a 22.1%–46.0% decrease in HR efficiency at 6 hours after CDK4/6i (Figure 2A). Using this reporter system, we examined HR repair in TNBC stable cell lines to determine if radiosensitization was due to changes in dsDNA break repair efficiency. In 2 separate MDA-MB-231 HR reporter clones, palbociclib, ribociclib, and abemaciclib pretreatment led to a significant decrease in HR efficiency (19.1%–58.7%) compared with vehicle-treated cells. The magnitude of HR suppression was similar to that of the CHK1/2 inhibitor AZD7762 (average decrease of 47.1% compared with vehicle), which is known to suppress HR activity and was used as a positive experimental control (30) (Figure 2, B and C). As an additional control, pretreatment with NU7441, a known inhibitor of DNA-dependent protein kinase (DNAPK) involved in nonhomologous end joining (NHEJ), led to an increase (average of 104.7% increase compared with vehicle) in HR efficiency by blocking NHEJ-mediated dsDNA repair. HR suppression did not occur in the RB-null cell line BT-549 with 500 nM CDK4/6 inhibitor pretreatment (Supplemental Figure 3, A and B).

To further understand the implications of CDK4/6 inhibitor–mediated radiosensitization and its effects on HR in a nonoverlapping model system, we quantified RAD51 foci formation after radiation using immunofluorescence microscopy. RAD51 is a recombinase that initiates the catalysis of HR-mediated DNA repair through involvement in the strand pairing process, and RAD51 foci formation is a surrogate measure for active HR activity (31). In MDA-MB-231 (Figure 2D), CAL-120 cells (Figure 2E and Supplemental Table 6), and SUM-159 cells (Supplemental Figure 3C) that express WT RB, irradiation with 4 Gy led to a sharp increase in the presence of RAD51 foci at both 6 and 16 hours after radiotherapy (average increase of 50.4%–67.54% compared with DMSO). However, in cells with a 1-hour palbociclib pretreatment, RAD51 foci at both 6 and 16 hours after radiotherapy significantly decreased compared with radiation alone (average decrease of 26.6%–31.56% at 6 hours and 18.1%–31.2% at 16 hours), indicating suppression of HR (Figure 2, D and E, and Supplemental Figure 3C). The decrease in RAD51 foci formation in MDA-MB-231, CAL-120, and SUM-159 cells was not explained by the absence of RAD51 protein, which remained constant at 6 hours after RT (Figure 2F and Supplemental Figure 3D), with only a modest decrease of RAD51 expression after palbociclib or combination treatment in the latest time point (16 hours).

In contrast to RB WT TNBC cell lines, MDA-MB-468 (Figure 2G) and CAL-851 TNBC cells (Figure 2H) that lack RB formed equivalent levels of RAD51 foci after RT, despite pretreatment with 1μM palbociclib (average of 46.3% positive in RT only cells and 53.0% in combination-treated cells at 6 hours). Thus, CDK4/6 inhibitor pretreatment did not lead to HR suppression at 6 or 16 hours (Supplemental Table 6). In these RB-null cell lines, RAD51 expression levels also remained relatively constant at both 6 hours and 16 hours after RT (Figure 2I). Representative immunofluorescence images of RAD51 foci are shown in both MDA-MB-231 cells and CAL-851 cells (Supplemental Figure 3, E and F) 6 hours after RT.

**Figure 3:**
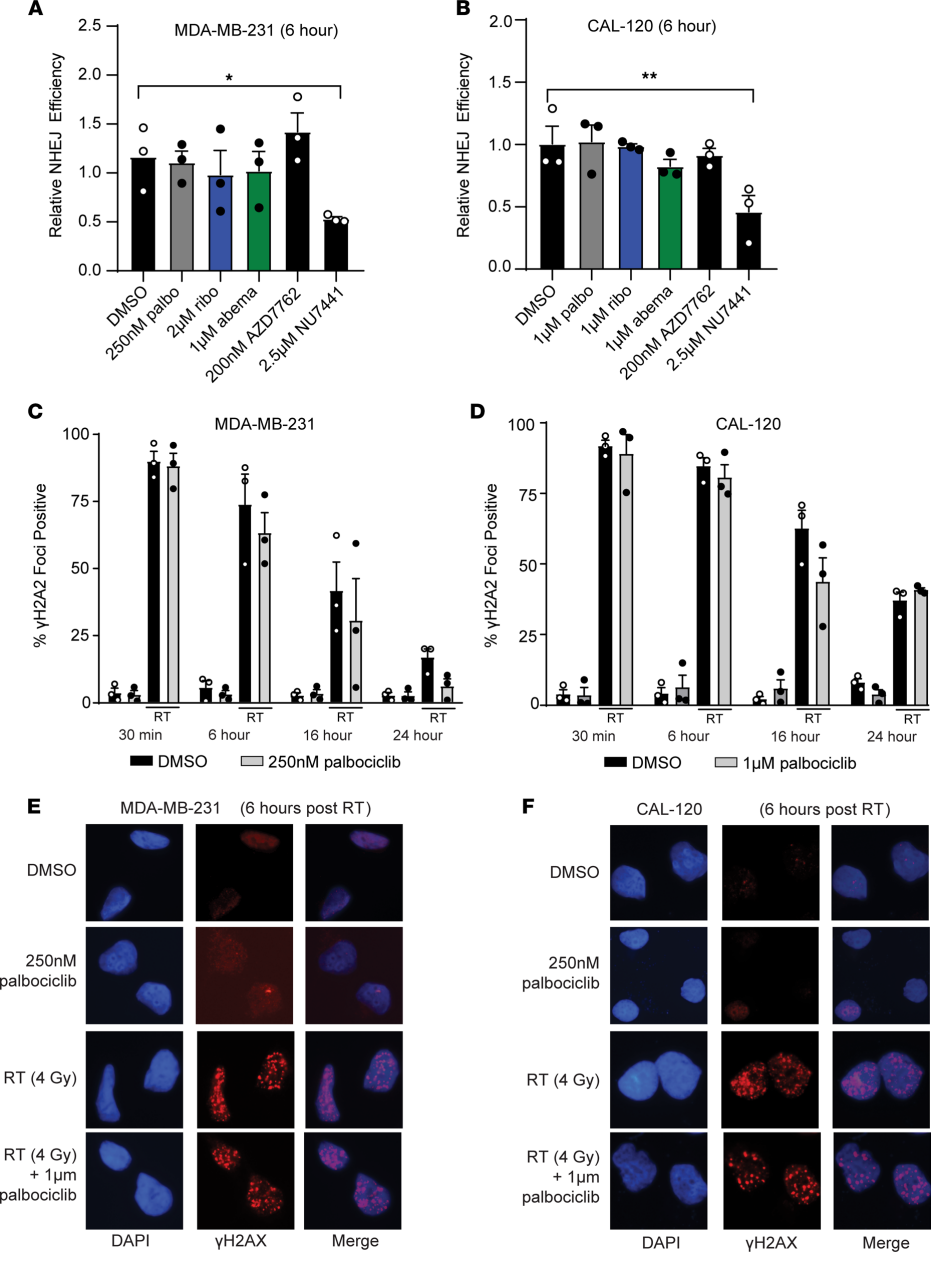
CDK4/6 inhibition does not suppress NHEJ efficiency. (**A** and **B**) A eYFP reporter system was used to measure relative NHEJ repair efficiency in RB WT MDA-MB-231 (**A**) and CAL-120 (**B**) cells after treatment with palbociclib (gray), ribociclib (blue), or abemaciclib (green). The CHK1/2 inhibitor (200 nM AZD7762) was used as a negative control, and the DNAPK inhibitor (2.5 μM NU7441) was used as a positive control. (**C**–**F**) Cells were fixed 0.5, 6, 16, and 24 hours after RT (2 Gy) in RB WT MDA-MB-231 (**C** and **E**) and CAL-120 (**D** and **F**) cells, and cells were stained for γH2AX foci (red) and DAPI (blue). *P < 0.05; **P < 0.01. All experiments represent the mean of *n =* 3 experiments. Two-tailed *t* tests were used to compare the RT and combination-treated groups at each γH2AX time point, and correction was performed for multiple comparisons. Original magnification, ×60.

To better understand the dynamic regulation of dsDNA break repair in our models, we used a transient pEYFP reporter system to assess the capacity of TNBC cell lines to conduct NHEJ-mediated DNA repair after CDK4/6i. Although we observed HR suppression 6 hours after CDK4/6i, treatment of RB WT MDA-MB-231 and CAL-120 TNBC cells with the IC_50_ concentration of palbociclib, ribociclib, or abemaciclib did not affect NHEJ efficiency after 6 hours (Figure 3, A and B). As expected, treatment with NU7441 (DNAPK inhibitor) significantly decreased NHEJ activity (average of 46.8% decreased compared with vehicle in MDA-MB-231 and 46.1% in CAL-120), but the CHK1/2 inhibitor AZD7762 (which inhibits HR) did not affect NHEJ repair efficiency in this system.

Next, we used immunofluorescence to measure γ-phosphorylation of histone 2AX (γH2AX foci) to assess the total number of dsDNA breaks and the kinetics of DNA repair in TNBC cell lines treated with RT and palbociclib. Consistent with previous literature (17, 26), CDK4/6i did not potentiate the number of total dsDNA or delay repair time in TNBC cell lines after radiation, remaining relatively constant at 0.5, 6, 16, and 24 hours after RT (Figure 3, C and D, and Supplemental Table 7). NHEJ is much faster than HR, and with CDK4/6i, the burden of dsDNA repair is increasingly shifted to error-prone NHEJ even prior to cell cycle arrest in G_1_. Representative images for γH2AX foci are shown in both MDA-MB-231 and CAL-120 cells (Figure 3, E and F).

### RB1 loss eliminates radiosensitivity to CDK4/6 inhibitors.

**Figure 4:**
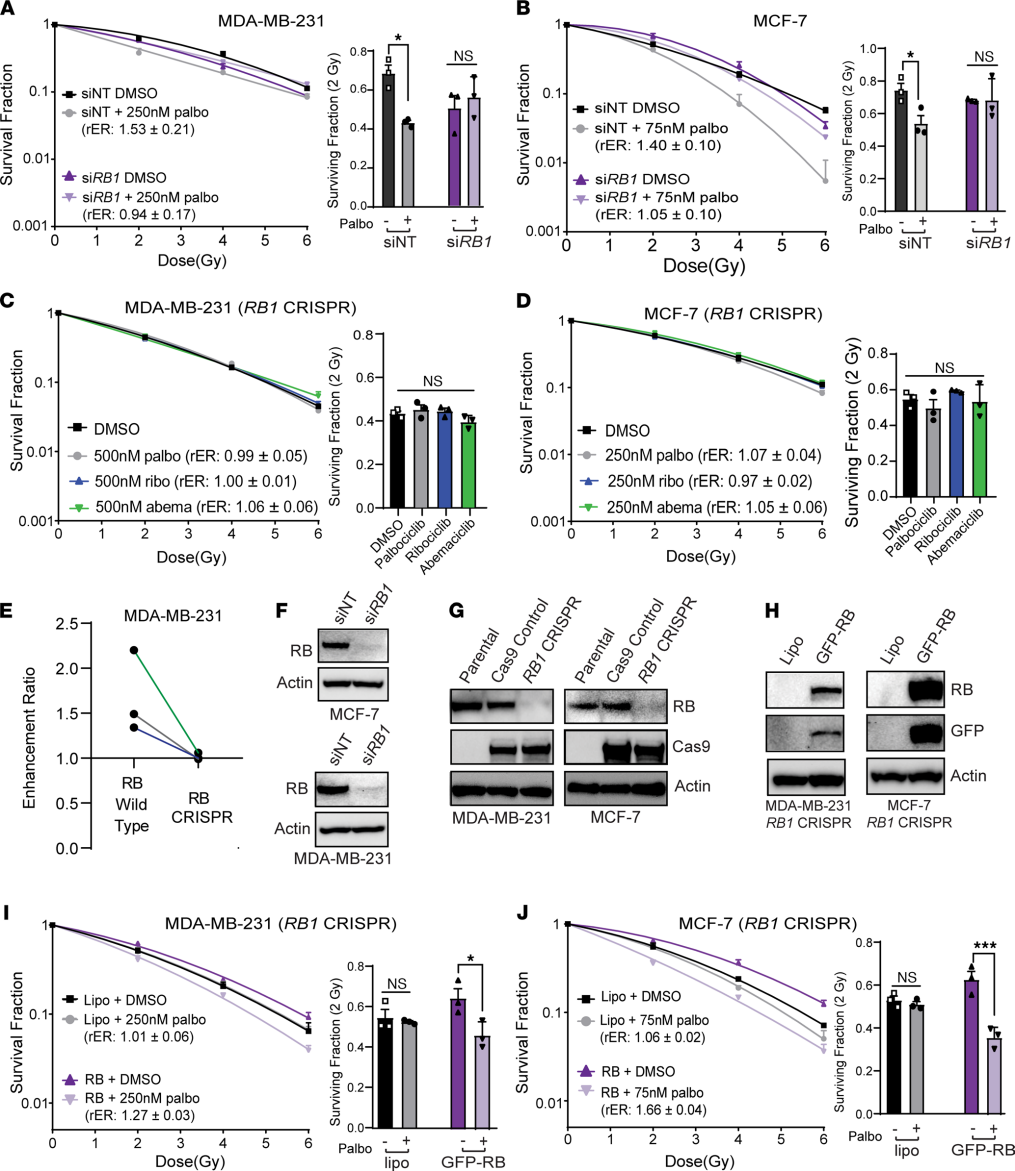
RB is required for the radiosensitization of TNBC cell lines. (**A**–**D**) Clonogenic survival assays were performed in breast cancer cell lines with transient knockdown of *RB1* (**A** and **B**) or CRISPR *RB1*-KO (**C** and **D**). (**E**) Average radiation enhancement ratios (rER) were compared between parental MDA-MB-231 cells and MDA-MB-231 *RB1* CRISPR cells. (**F**–**H**) The efficiency of siRNA mediated knockdown (**F**), CRISPR *RB1*-KO (**G**), and RB overexpression (**H**) were assessed using Western blots, where transfected samples were harvested 48 hours after transfection. (**I** and **J**) RB was transiently overexpressed in MDA-MB-231 (**I**) and MCF-7 (**J**) *RB1* CRISPR cell lines, and clonogenic survival assays were used to assess rescue of the radiosensitization phenotype. For CRISPR cells, a 1-way ANOVA with Dunnett’s post hoc test was used to compare CDK4/6 inhibitor–treated cells against vehicle-treated cells. For transfected cells, treatment pairs were compared with a 2-tailed Student’s *t* test and corrected for multiple comparisons. *P < 0.05; ***P < 0.001. All clonogenics represent the average of 3 experiments and are graphed as average ± SEM; Western blots are representative blots from *n =* 3 experiments.

To investigate whether intact RB protein expression was necessary for radiosensitization in breast cancer cell lines, we first sought to determine how *RB1* loss would affect the radiation response in breast cancer cell lines. CDK4/6 inhibitor–mediated radiosensitization was preserved in control siNT cells treated with palbociclib and RT (MCF-7 rER: 1.40 ± 0.10, MDA-MB-231 rER: 1.53 ± 0.21) compared with cells treated with RT alone (Figure 4, A and B). Transient knockdown of *RB1*, however, abolished palbociclib-mediated radiosensitization of MCF-7 (rER: 1.05 ± 0.10) and MDA-MB-231 (rER: 0.95 ± 0.17) cells compared with si*RB1*-transfected cells treated with vehicle. Notably, transient loss of RB expression was associated with an overall increase in the intrinsic radiosensitivity of breast cancer cells — particularly in MDA-MB-231 cells.

To further confirm these studies, we generated isogenic models of RB loss in ER^+^ and TNBC cell lines utilizing the CRISPR-Cas9 system. Loss of RB in MDA-MB-231, CAL-120, MCF-7, and T47D cells led to a decrease in potency — but not a complete absence of effect — for all 3 CDK4/6 inhibitors as monotherapies (Supplemental Table 1 and Supplemental Figure 4). Control cell lines expressing the Cas9 protein did not show significant differences in the IC_50_ value compared with parental cell lines.

RB KO also affected CDK4/6 inhibitor–mediated cell cycle arrest in *RB1*-KO cell lines. In parental cell lines that express RB (CAL-120, MDA-MB-231; Supplemental Figure 5, A and D), treatment with a CDK4/6 inhibitor led to cell cycle arrest in G_1_ after 24–48 hours. Although Cas9 control cells retained the ability to undergo CDK4/6 inhibitor–mediated G_1_ arrest (Supplemental Figure 5, B, E, and H), ER^+^ and TNBC *RB1*-KO cell lines fail to arrest in G_1_ after 24- to 48-hour treatment with palbociclib, ribociclib, or abemaciclib (Supplemental Figure 5, C, F, and I). These results are consistent with the RB-null cell line MDA-MB-468, which also fails to arrest in G_1_ phase after CDK4/6 inhibitor treatment (Supplemental Figure 5G).

To determine the effect of RB protein levels on radiosensitivity in isogenic models, we performed clonogenic survival assays. We observed a complete loss of radiosenstization in MDA-MB-231 (rER: 0.99–1.06; Figure 4C) and CAL-120 *RB1* CRISPR cells (rER: 0.94–1.13; Supplemental Figure 6A), despite using higher concentrations of palbociclib, ribociclib, or abemaciclib compared with those used to treat parental TNBC cell lines. Similar results were obtained in the ER^+^ cell lines MCF-7 and T47D (Figure 4D and Supplemental Figure 6B; rER: 0.88 – 1.07) where we failed to observe a concentration-dependent increase in radiosensitization or a decrease in the surviving fraction of cells at 2Gy. As expected, Cas9-expressing control cell lines retained palbociclib-mediated radiosensitization at levels comparable with the respective parental cell lines (rER: 1.26–1.60; Supplemental Figure 6, C and D). When compared with WT MDA-MB-231 and CAL-120 cells, mean rER values for each CDK4/6 inhibitor significantly decreased in MDA-MB-231 and CAL-120 CRISPR *RB1*-KO cells (Figure 4E and Supplemental Figure 6F), consistent with RB-dependent radiosensitization. In *RB1*-KO cells, Western blots were used to confirm successful KO of RB at the protein level (Figure 4, F–H, and Supplemental Figure 6E).

In order to test if reexpression of RB was sufficient to rescue the radiosensitization phenotype, we engineered a Cas9-resistant RB expression plasmid using site-directed mutagenesis of a GFP-tagged RB plasmid. By creating 4 synonymous mutations in the coding sequence recognized by the CRISPR gRNA, we were able to overexpress RB protein in our ER^+^ and TNBC models of *RB1* loss without changing the amino acid sequence of the RB protein. Reexpression of RB protein in *RB1* CRISPR cells was radioprotective, leading to an increase in the surviving fraction of cells at 2 Gy and an upward deflection of the surviving fraction curve (solid purple). After reintroduction of RB, radiosensitization was restored after palbociclib pretreatment (MCF-7 rER: 1.66 ± 0.04, MDA-MB-231 rER: 1.27 ± 0.03) (Figure 4, I and J). In addition, transient expression of RB protein in MDA-MB-468 or BT-549 cells led to modest radiosensitization (rER: 1.27–1.36) (Supplemental Figure 6, G–I), though we only tested this effect up to a concentration of 1 μM palbociclib.

We also engineered models of RB loss through serial culture of MDA-MB-231 cells in palbociclib-containing media to create CDK4/6 inhibitor–resistant MDA-MB-231 subclones (MDA-MB-231 palbociclib-resistant cells [PalboR]; Supplemental Figure 7A, IC_50_ > 5μM for all CDK4/6 inhibitors). These cells demonstrated decreased sensitivity to all 3 CDK4/6 inhibitors, and similar to MDA-MB-231 *RB1* CRISPR clones, MDA-MB-231 PalboR cells demonstrated a loss of RB protein. In clonogenic survival assays, MDA-MB-231 PalboR cells were not radiosensitized with palbociclib, ribociclib, or abemaciclib pretreatment (rER: 0.97–1.09; Supplemental Figure 7B). To reaffirm the RB dependence of radiosensitivity to CDK4/6i, we transiently expressed the GFP-RB protein in MDA-MB-231 PalboR cells, which rescued palbociclib-mediated radiosensitization (Supplemental Figure 7C).

Because there are data to suggest that mutations in other tumor suppressor proteins — such as *TP53* — may play a role in response to CDK4/6 inhibitor monotherapy or combination therapy (32), we generated additional isogenic models of *TP53* loss in *TP53* WT ER^+^ (MCF-7) and TNBC (CAL-51) cell lines (Supplemental Figure 8, A and B) and performed clonogenic survival assays. Radiosensitization was maintained in CAL-51 and MCF-7 *TP53* CRISPR cells (Supplemental Figure 8, C and D) compared with Cas9 controls (Supplemental Figure 8E and Supplemental Figure 6D), though the magnitude of the effect was slightly diminished at the highest concentrations used. Nonetheless, these findings corroborate earlier data in p53 mutant parental TNBC cell lines (Figure 1, D–G), suggesting that *TP53* status does not predict response to CDK4/6i + RT.

### CDK4/6i radiosensitizes RB-expressing TNBC tumors in vivo.

**Figure 5:**
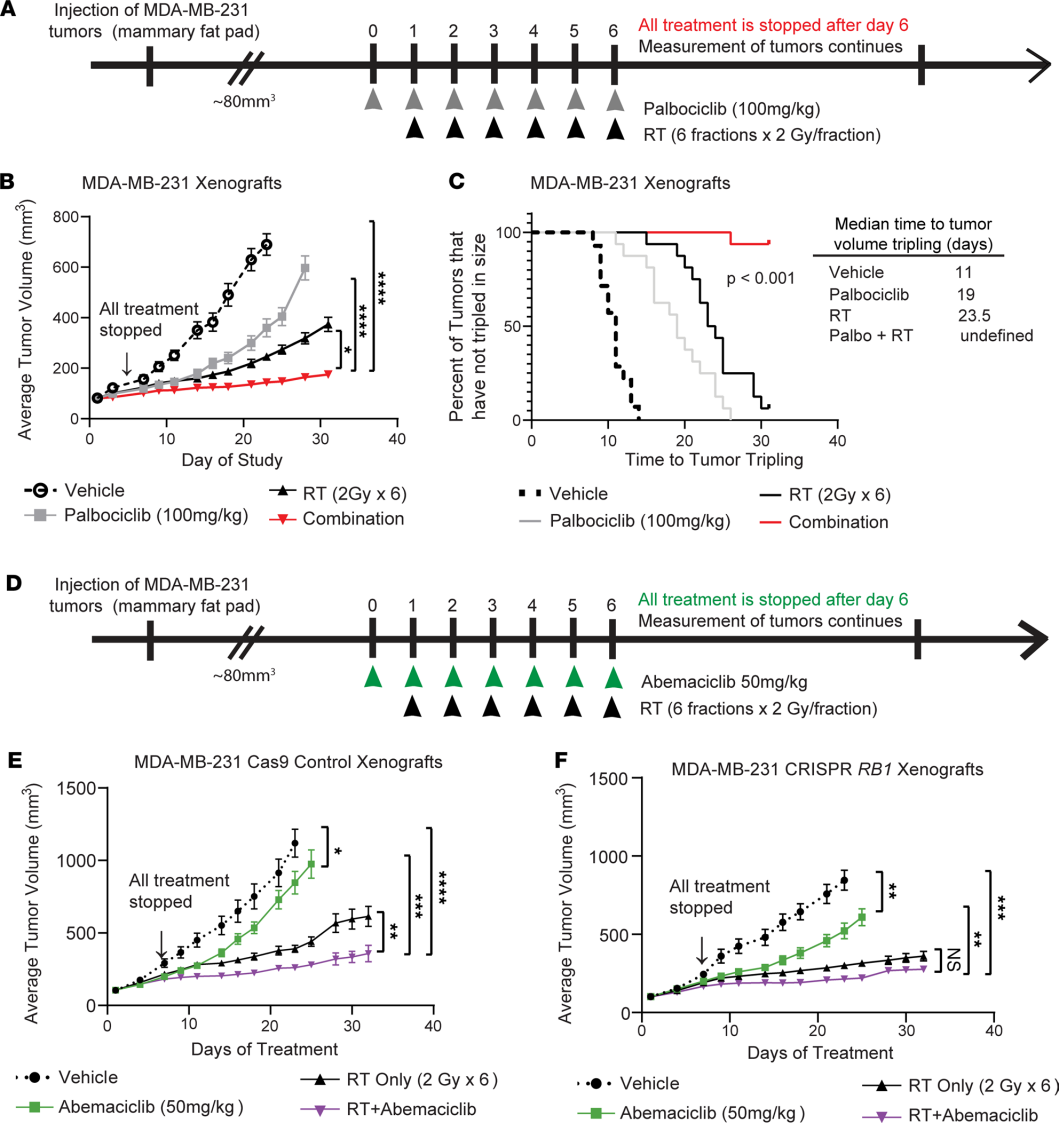
CDK4/6 inhibition radiosensitizes TNBC cells in vivo. (**A**) Mice bearing parental (RB WT) MDA-MB-231 xenografts were randomized to 4 treatment groups (8–9 tumors per group): vehicle (dashed lines, open circles), RT only (solid black, triangles), 100 mg/kg palbociclib (gray, squares), and the combination (red, inverted triangles). (**B** and **C**) Tumor size was measured 2–3 times a week and used to calculate average tumor volume (**B**; graphed as average ± SEM) and time to tumor tripling (**C;** log-rank Mantel-Cox test). (**D**–**F**) Xenografts with CRISPR MDA-MB-231 cells (**D**) expressing either Cas9 only (**E**) or Cas9 and the *RB1* guide RNA (**F**) were treated in a similar treatment schedule using 50 mg/kg abemaciclib (green, squares) or the combination of abemaciclib + RT (purple, inverted triangles). Tumor volume was compared using a 1-way ANOVA comparing average tumor size at the end of the study across the treatment groups. *P < 0.05; **P < 0.01; ***P < 0.001, ****P < 0.0001.

To characterize the effects of combined CDK4/6i and radiation in an in vivo model, we generated xenografts using MDA-MB-231 cells injected into the mammary fat pads of female mice (Figure 5A). Mice received either vehicle, 100 mg/kg palbociclib alone, radiation alone, or a combination of palbociclib and radiation. Palbociclib treatment in the combination group was started 1 day prior to the start of radiotherapy that was delivered in 6 fractions at 2 Gy/fraction; all treatment was halted after the last fraction of radiation. Combination treatment with palbociclib and RT significantly suppressed tumor growth in mice compared with treatment with RT or palbociclib alone (Figure 5B). There was a significant delay in time to tumor doubling (Supplemental Figure 9A, *P <* 0.001) and tripling (Figure 5C, *P <* 0.001) in the combination-treated group (undefined) compared with tripling times for mice treated with vehicle (11 days), palbociclib alone (19 days), or radiation (23.5 days). Short-term treatment of mice with palbociclib and RT demonstrated suppression of Ki67 staining in combination-treated groups, demonstrating on-target effects of palbociclib and RT (Supplemental Figure 9, C and D).

To elucidate the RB-dependence of CDK4/6 inhibitor–mediated radiosensitization in vivo, we utilized isogenic MDA-MB-231 xenograft models expressing either Cas9 alone or Cas9 and the *RB1* CRISPR guide (Figure 5D). In Cas9-expressing xenografts, RT and abemaciclib (50 mg/kg) demonstrated modest single-agent efficacy, but combined abemaciclib + RT led to significant radiosensitization in vivo (Figure 5E, *P <* 0.01). Although *RB1* CRISPR xenograft tumors (Figure 5F) were not radiosensitized by abemaciclib + RT (*P >* 0.05), *RB1* CRISPR xenografts demonstrated increased single-agent sensitivity to RT, consistent with the proposed role of RB in responding to DNA damage. Despite *RB1* KO, modest single-agent effects of abemaciclib were observed — likely due to the effects of abemaciclib on other CDKs such as CDK5 and CDK9.

Overall, all treatments were tolerated without significant weight loss (Supplemental Figure 9, B, E, and F). Synergy between CDK4/6i with palbociclib (Supplemental Table 4) and abemaciclib (Supplemental Table 5) and radiation was calculated, as described previously (33). Combination treatments were synergistic — not just additive — in RB-expressing xenografts by the end of the study (parental and Cas9 control xenografts; ratio > 1), which did not occur in *RB1* CRISPR xenografts (Supplemental Table 5), as expected.

### RB is involved in HR-mediated dsDNA repair.

To assess whether or not changes in the intrinsic sensitivity of *RB1* CRISPR cells was mediated through aberrant mitosis and increased mitotic catastrophe, we performed immunofluorescence to quantify micronuclei formation in MCF-7 and MDA-MB-231 cells 72 hours after 4 Gy RT (Supplemental Figure 10) (34). As expected, in *RB1* WT parental cells, radiation led to an increase in micronucleated cells (23.1% in MDA-MB-231, 26.0% in MCF-7 cells). However, RT-induced micronuclei formation was not potentiated in the *RB1* CRISPR cell lines and occurred at similar magnitudes (16.9% increase in MDA-MB-231 *RB1* CRISPR cells after RT and 22.9% increase in MCF-7 *RB1* CRISPR cells) to the parental cell lines.

**Figure 6:**
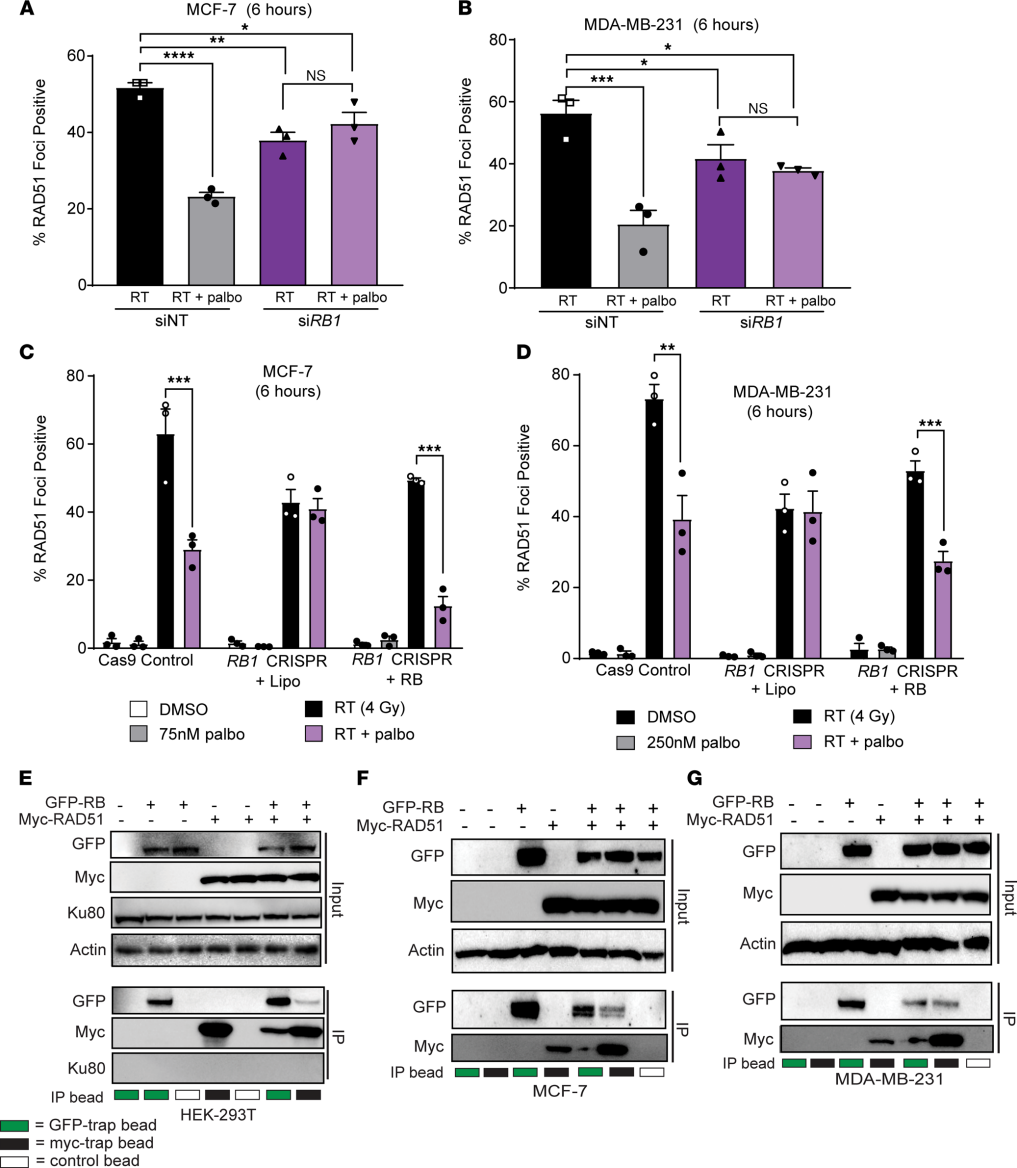
RB is required for efficient repair of dsDNA breaks through HR. (**A**–**D**) RAD51 foci formation was used to assess HR competency with transient (**A** and **B**) knockdown or CRISPR-KO (**C** and **D**) of *RB1* in MCF-7 and MDA-MB-231 cells at 6 hours after radiation. Cells transfected with a control siRNA or cells expressing Cas9 with control guides (*AAVS1*) were used for comparison. (**E**–**G**) Immunoprecipitation of GFP-RB and myc-RAD51 was performed 24 hours after transfection of HEK-293T cells (**E**), MCF-7 cells (**F**), or MDA-MB-231 cells (**G**) using myc-trap beads (black boxes), GFP-trap beads (green boxes), or control beads (white boxes). Expression of myc-RAD51 and GFP-RB was assessed in protein inputs and IP lysates with Western blotting. In siRNA transfected cells, a 1-way ANOVA was used to compare treatment groups. For CRISPR experiments performed across multiple cell lines, 2-tailed *t* tests were performed between paired radiation, and combination-treated groups within each cell line and correction was performed for multiple comparisons. *P < 0.05; **P < 0.01; ***P < 0.001, ****P < 0.0001. All experiments represent the mean of *n =* 3 independent experiments.

To further elucidate the effects of *RB1* loss in ER^+^ and TNBC cell lines, we quantified RAD51 foci formation after transient *RB1* knockdown or CRISPR-mediated *RB1* loss. In MCF-7 and MDA-MB-231 cells, transient RB loss resulted in a decrease in the overall induction of RAD51 foci following radiation (4 Gy) treatment (average of 53.9% positive in siNT cells after RT and 37.8% positive in si*RB1* cells after RT; Figure 6, A and B, and Supplemental Table 8). While control cells demonstrate CDK4/6 inhibitor–mediated suppression of RAD51 foci formation, indicative of HR suppression, si*RB1* cells do not demonstrate suppression of RAD51 foci formation after palbociclib treatment.

Consistent with models of transient *RB1* knockdown, MCF-7 and MDA-MB-231 *RB1* CRISPR cells displayed global suppression of RAD51 foci formation compared with Cas9 control cell lines (average of 68.2% positive in Cas9 cells after RT and 42.6% positive in *RB1* CRISPR cells after RT; Figure 6, C and D). Overexpression of RB protein in *RB1* CRISPR cells increased the overall quantity of radiation-induced RAD51 foci and increased susceptibility of TNBC and ER^+^ cells to CDK4/6 inhibitor–mediated HR suppression (Figure 6, C and D). Representative images of RAD51 foci are shown 6 hours after RT for MCF-7 and MDA-MB-231 cells transfected with si*RB1* (Supplemental Figure 11, A and B), as well as MCF-7, MDA-MB-231 Cas9, and *RB1* CRISPR cells (Supplemental Figure 11, C–E, and Supplemental Table 9).

With a clear decrease in HR activity (RAD51 foci) driven by RB-dependent CDK4/6 inhibitor–mediated radiosensitization, we next sought to determine the molecular mechanism linking RB to HR. Thus, we cotransfected HEK-293T cells with GFP-RB or Myc-tagged RAD51 and performed immunoprecipitation 24 hours after transfection (Figure 6E). Pulldown of GFP-RB also resulted in pulldown of myc-RAD51 protein, and the reverse occurred with pulldown of myc-RAD51, resulting in pulldown of GFP-RB protein. In contrast, the NHEJ mediator Ku80 was not pulled down with either GFP-RB pulldown or myc-RAD51 pulldown, suggesting that it may not be necessary for RB-dependent repair of dsDNA breaks. This interaction between GFP-RB and myc-RAD51 was also observed in the ER^+^ breast cancer cell line MCF-7 (Figure 6F) and the TNBC cell line MDA-MB-231 (Figure 6G). To control for nonspecific effects of overexpression, we also performed immunoprecipitation with GFP-RB and myc-MCL1, a mitochondria-associated protein involved in apoptosis, and we did not observe nonspecific pulldown of GFP-RB as an artifact of overexpression (Supplemental Figure 11F).

## Discussion

In this study, we demonstrate that CDK4/6i and radiotherapy led to an RB-dependent increase in both radiosensitization and breast cancer cell death in multiple, nonoverlapping ER^+^ and TNBC models in vitro (Figure 1) and in vivo (Figure 5). Cells treated with a CDK4/6 inhibitor in the presence of RB were radiosensitized through cell cycle–independent impairment of HR activity and not NHEJ (Figure 3); neither radiation sensitivity nor HR activity was affected in RB-null TNBC cell lines (Figure 2). The blockade of CDK4/6 inhibitor–mediated radiosensitization produced by transient knockdown or complete KO of *RB1* could be rescued with transient overexpression of RB protein (Figure 4), further confirming the key role of RB in radiosensitization. Finally, our findings suggest that RB may affect HR by interacting with key protein members of HR repair (such as RAD51) in breast cancer cell lines (Figure 6). These results demonstrate that the combination of CDK4/6i and radiotherapy is a potentially effective strategy not just for ER^+^ breast cancers, but for the radiosensitization of other *RB1* WT cancers including TNBC, which have disproportionately high rates of locoregional recurrence after RT.

Although the role of RB in cell cycle progression through the G_1_/S checkpoint is well characterized, recent reports have implicated RB expression as a necessity for promoting efficient DNA damage response in cancer cells (35). RB expression can be used to predict sensitivity of cancer cells to antimitotic and cytotoxic chemotherapies (12, 16), and recent evidence suggests that CDK4/6 inhibitors can be used to suppress the DNA damage response in RB-dependent cancers (24). Finally, the ataxia telangiectasia mutated (ATM) protein, which sits at the apex of dsDNA break repair, has been shown to phosphorylate RB (36) or E2F1 (27), leading to RB-dependent repair of dsDNA breaks.

The role of RB in DNA damage repair has been implicated in both NHEJ-mediated (37) and HR-mediated repair (27, 38). CDK4/6 inhibitor–mediated G_1_ arrest in TNBC cell lines shifts the DNA damage response away from HR to the more error-prone dsDNA repair pathway, NHEJ, resulting in increased DNA damage (11, 26). Additionally, loss of RB may modulate expression of γH2AX, a measure for the total number of dsDNA breaks, in cancer cell lines (39, 40). Although we were unable to reproduce the finding that RB protein forms foci after radiation that are colocalized with γH2AX at dsDNA break sites (27), we instead demonstrated that RB interacts within the same protein complex as RAD51 in the process of HR-dependent DNA repair. Although further studies are needed to determine the complex dynamics of RB and RAD51 interaction and the time course of recruitment for additional DNA damage response proteins, we have demonstrated here that manipulation of RB expression changes both the radiosensitivity of breast cancer cells and their susceptibility to CDK4/6 inhibitor–mediated suppression of HR. Specifically, we show that the absence of RB impairs the recruitment of RAD51 to sites of dsDNA damage (resulting in less RAD51 foci), but overexpression of GFP-RB can increase RAD51 recruitment (more RAD51 foci).

There is a growing body of evidence suggesting that other cyclin-dependent kinases including CDK7, CDK8, CDK9, and CDK12 may also be important for modulating the sensitivity of cancer cells to the effects of ionizing radiation (41–43). In contrast to CDK4/6 inhibitor–mediated radiosensitization in breast cancer that is primarily mediated through suppression of HR, inhibition of other CDK proteins leads to increased apoptosis, senescence, and/or inhibition of RNA Polymerase II function that leads to radiosenstization (41–43). Furthermore, specific inhibition of CDK9 may be mediated through suppression of Axl-mediated signaling pathways (44). In our study, abemaciclib — which inhibits other CDK proteins including CDK9 — was consistently the most potent and effective radiosensitization agent compared with palbociclib and ribociclib, which are more selective for CDK4/6. Although pan-CDK inhibitors such as roscovitine, flavorpiridiol, and roniciclib have also demonstrated potential as radiosensitization agents in a variety of cancer types, targeted inhibition of individual CDK proteins provides a more targeted therapeutic approach with greatly reduced side effect profiles (45–50). Future studies are needed in this setting to explore if there are additional mechanisms — beyond HR suppression — that could be exploited with the use of CDK inhibition + RT in ER^+^ breast cancer and TNBC.

CDK4/6 inhibitors may be administered with other types of therapies — particularly endocrine therapies in ER^+^ disease or novel immune checkpoint inhibitors in TNBC — which may influence cell survival when administered concurrently with radiation. CDK4/6i has been shown to promote antitumor immunity (51–54), leading to greater immunogenicity of TNBC cells in vivo. Radiation is known to stimulate the immune system in breast cancer (55, 56), and immunotherapies have recently been approved for use in TNBC (57). Many combination treatments with CDK4/6i and immune checkpoint inhibitors (targeting PD-1 and CTLA-4) have been proposed (58), but the effects of CDK4/6i on radiation-induced antitumor immunity — and the potential for those effects to be altered in the presence of immune checkpoint inhibitor therapy — have yet to be elucidated and are an important future direction of this work.

CDK4/6 inhibitors are currently only approved to treat ER^+^ breast cancers, but our data build on a growing body of evidence that suggest there may be clinical relevance in expanding the use of CDK4/6i in combination with DNA damaging or cytotoxic agents to treat TNBC and other cancer types. Although the rates of RB mutation are higher in TNBC (7%; ref. 20), the RB dependence of CDK4/6 inhibitor–mediated radiosensitization may be an important consideration in patient selection for future clinical trials in both ER^+^ breast cancer and TNBC. Furthermore, in additional cancers such as oral squamous cell carcinoma and hepatocellular carcinoma, palbociclib significantly inhibits cellular growth, accelerates senescence and apoptosis, and suppresses RAD51 foci formation (59, 60). Thus, CDK4/6i + RT may be a viable therapeutic strategy in other cancer types.

We have demonstrated the potential for radiosensitization of TNBC models utilizing all 3 of the clinically approved CDK4/6 inhibitors, but in future studies, we will evaluate whether this strategy is effective in other histopathological classifications or subtypes of breast cancer, such as inflammatory breast cancer, lobular breast cancer, and HER2-enriched breast cancer. In addition, future studies are needed to address optimal sequencing of drug and radiation in both ER^+^ and TNBC in order to optimize treatment efficacy (18). These studies will determine whether pretreatment, concurrent treatment, or continued adjuvant treatment with drug is most effective in the context of RT treatment, and these studies will be critical to phase I/II clinical trial design in women with RB intact breast cancers at high risk for locoregional recurrence. Finally, animal studies utilizing patient-derived xenograft (PDX) models will help offer additional compelling evidence that combinatory treatment with CDK4/6i and radiotherapy may be a clinically relevant strategy for TNBC.

Taken together, our results suggest that CDK4/6i + RT is a promising strategy to decrease recurrence and increase local disease control across ER^+^ and TNBC and suggest that RB may be a potential biomarker for efficacy. Concerns about the safety of concurrent CDK4/6i and RT remain, and to date, small studies exploring outcomes in patients with metastatic breast cancer receiving palliative radiation and CDK4/6 inhibitor therapy have conflicting results regarding toxicity, depending on the dose/fractionation of radiation and the regions targeted in the metastatic setting (visceral organs; refs. 61–64). Reassuringly, to our knowledge, no studies to date have demonstrated more pronounced side effects of CDK4/6 inhibitors, such as cytopenias and skin desquamation, in women receiving concurrent therapy to the breast or axillary regions. To address this issue directly, we have developed a phase I trial to assess the safety, tolerability, and preliminary efficacy of this combination in women with multinode-positive ER^+^ breast cancer, which is opening soon. In our proposed study, all subjects would receive breast/chest wall and regional nodal therapy with highly conformal techniques designed to minimize dose to adjacent organs. We do not anticipate that concurrent radiation therapy and CDK4/6i will exacerbate the known potential toxicities of these agents; however, the effect of CDK4/6i on skin toxicities during postoperative RT will be carefully assessed on study.

Provided that the combination therapy is well tolerated, these data will also inform the planned phase II randomized clinical trial evaluating the efficacy of combined CDK4/6i and RT in women with multiple lymph node–positive (LN^+^) ER^+^ breast cancer (>3 LN). The preclinical studies described herein provide the rationale for expanding the eligibility for these trials into women with RB intact TNBC for whom locoregional control remains a significant clinical issue. Finally, this proposed combination therapy has the potential to provide an effective “targeted therapy” for the treatment of TNBC with radiation where no such targeted therapy currently exists.

## Methods

### Cell culture.

Cell lines from ATCC were expanded from frozen stocks and maintained at 37°C and 5% CO_2_. T47D, MCF-7, MDA-MB-231, MDA-MB-468, and CAL-120 cells were grown in DMEM (Thermo Fisher Scientific, 11965-092) with 10% FBS (Atlanta Biologicals). BT-549 were grown in RPMI, and CAL-851 cells were grown in DMEM (Thermo Fisher Scientific, 11995-040) with 10% FBS. SUM-159 cells were grown in HAMS F-12 (Thermo Fisher Scientific, 11765-054) with 5% FBS, 0.01M HEPES (Thermo Fisher Scientific, 15630080), 6 μg/mL insulin (MilliporeSigma, I9278), and 1 μg/mL hydrocortisone. All cell lines were supplemented with 5% penicillin/streptomycin (Invitrogen, 15140122) and used for experiments at subconfluent densities. Cell lines were authenticated with STR profiling, and mycoplasma testing (Lonza, LT07-701) was performed monthly. PalboR cells were generated by culturing MDA-MB-231 cells in dose-escalating concentrations of palbociclib (50 nM to 1 μM) over a period of 3 months.

### Drugs.

Palbociclib (MilliporeSigma, PZ0199), abemaciclib (Med Chem Express, HY-16297A), and ribociclib (Med Chem Express, HY-15777A) were used to make 10 mM stocks in 100% DMSO for in vitro assays. NU7441 (SelleckChem, S2638) and AZD7762 (SelleckChem, S1532) were also obtained commercially in 10 mM DMSO.

### Clonogenic survival assays.

Cells plated at single-cell density were pretreated with CDK4/6 inhibitor 1 hour prior to RT. Plates were radiated (0–6 Gy) and returned to the incubator for 1–3 weeks before methanol and acetic acid fixation (7:1) and staining with crystal violet (1%). Drug-containing media remained on the cells during the incubation phase, but the drug was not replaced or replenished at any time after the initial day of treatment. Colonies were defined as 50 or more cells and counted from each treatment condition to calculate the surviving fraction for each treatment group. Survival curves were calculated as described previously (33, 65), and enhancement ratios were calculated by taking a ratio of the area under each of the surviving fraction curves, with the area for the control (RT only) condition divided by the AUC for each experimental condition.

### Immunofluorescence.

A total of 100,000 cells was plated onto coverslips in 12-well plates and treated the next morning with either palbociclib or vehicle (DMSO) 1 hour prior to radiation (4 Gy). Coverslips were fixed at 6 hours or 16 hours after RT (4 Gy) for RAD51, and γH2AX foci were fixed at 0.5, 6, 16, and 24 hours after RT (2 Gy). Cells were fixed in 4% paraformaldehyde (Thermo Fisher Scientific, J19943K2) with 2% sucrose (MilliporeSigma, S9378) and 0.2% Triton X-100 (MilliporeSigma, T8532), permeabilized with 0.5% Triton X-100, and blocked in 1× PBS containing 5% goat serum (Thermo Fisher Scientific, 16210064), 0.5% BSA, and 0.05% Triton X-100. The phospho-histone H2AX (S139) monoclonal antibody (MilliporeSigma, 05-636, 1:2000) or the anti-RAD51 antibody (GeneTex, GTX70230 1:300) were used with the goat anti–mouse fluorescent secondary antibody (Invitrogen, A11005, 1:2000) to stain foci. A minimum of 100 cells were used to score and analyze formation of γH2AX and RAD51 foci. To quantify the exact number of foci per cell, ImageJ (NIH) was used to count image maxima. Cells with more than 15 γH2AX foci or more than 10 RAD51 foci were scored as positive. To quantify micronuclei formation, cells were radiated at 4 Gy and fixed in 4% paraformaldehyde after 72 hours before mounting directly on coverslips with DAPI stain (34).

### Immunoblotting.

After treatment, cells were washed using PBS and lysed using RIPA buffer (Thermo Fisher Scientific, 89901) with protease and phosphatase inhibitors (MilliporeSigma, PHOSS-RO and CO-RO). Western blotting was done using anti-RB (Cell Signaling Technology [CST], 9313S), anti-GFP (CST, 2956S), anti–phospho-RB (Ser807/811; CST, 8516 or Ser780; CST, 8180), anti-Cas9 (CST, 14697S), anti-myc (MilliporeSigma, 05419), anti-RAD51 (MilliporeSigma, ABE257), and anti-p53 (CST, 2524) antibodies. All primary antibodies were diluted at a 1:1000 dilution in 1% milk; HRP-conjugated β-actin (CST, 12262S) was diluted 1:50,000. Visualization was performed using 1:10,000 HRP-conjugated secondary antibodies (CST, 7074S and 7076S) and ECL prime (Cytiva, RPN2236). RB expression was quantified using ImageJ, normalized to β-actin expression for each lane, and calculated relative to the median RB expression of the blot.

### Xenograft studies.

In total, 2 × 10^6^ WT, *RB1* CRISPR, or Cas9 control MDA-MB-231 cells were injected into the mammary fat pads of 6- to 8-week-old CB17-SCID female mice (obtained from a University of Michigan colony originally sourced from Charles River Laboratories) with Matrigel (Thermo Fisher Scientific, CB-40234) with 14–16 tumors per group. Once tumor volume reached 80–100 mm^3^, mice were randomly assigned to either vehicle treatment, drug alone, radiation alone, or the combination. CDK4/6 inhibitor was given by oral gavage with either palbociclib (100 mg/kg) in 50 mmol/L (pH 4.0) sodium L-lactate (MilliporeSigma, L-7022) or abemaciclib (50mg/kg) in 25 mM phosphate buffer pH 2.0 (MilliporeSigma, 09568) with 1% hydroxyethylcellulose. Mice in the combination groups were treated 24 hours prior to the first RT dose and dosed concurrently with radiation for 5 days; treatment for all groups was stopped at day 6. Tumor growth was measured using calipers, and tumor volume was calculated using the equation *V* = (*L* × *W*2) × π/6. For short-term studies, mice were treated with palbociclib 1 day (24 hours) prior to RT and continued concurrently with RT (1 hour pretreatment each day) for 2 days. Tumors were harvested 1 hour after the last fraction of RT (2 fractions × 2 Gy), and IHC was performed with the help of the University of Michigan Research Histology and Immunohistochemistry Core. Ki67 slides were imaged at 40×, and 5 high-powered fields from 4 tumors (20 total) were quantified per treatment condition using ImageJ (NIH).

### HR repair efficiency assay.

The HR DR-GFP reporter plasmid transfected into cell lines and, after 48 hours, selection of stable clones was performed with geneticin (Thermo Fisher Scientific, 10131027). Flow cytometry was used to sort for GFP^+^ cells, and verified single clones were expanded. Cells were plated and pretreated with CDK4/6 inhibitor, 1.5 μM NU7441, or 200 nM AZD7762 for 1 hour, after which SceI adenovirus was added for 48 hours to induce dsDNA breaks. Cells were then harvested, ethanol-fixed, and analyzed using flow cytometry to detect GFP^+^ cells.

### Transfections and siRNA.

Pooled siRNA guides targeting *RB1* (Dharmacon, L-003296-02) and control siRNA (Dharmacon, D-001810-10) were used at a stock concentration of 20 μM and a final concentration of 25 nM. Lipofectamine 2000 (Thermo Fisher Scientific, 11668030) was used to transfect cells in Opti-MEM (Invitrogen, 31985-062) and antibiotic-free media. Transfected cells were replated for in vitro assays 24 hours after transfection and treated with CDK4/6 inhibitor and/or RT 48 hours after transfection.

### CRISPR.

The lentiCRISPRv2 plasmid (Addgene, 98290) was digested with BsmBI for 15 minutes at 55°C and gel-purified using the QIAquick Gel Extraction Kit (Qiagen, 28706X4). Oligos from IDT were annealed at 95°C cooled at 5°C/minute. The *RB1* guide sequence (5′ CACCGGGTTCTTTGAGCAACATGGG 3′) or the *TP53* guide sequence (5′ CACCGCCATTGTTCAATATCGTCCG 3′) was ligated into the CRISPR plasmid and transformed into Stbl3 bacteria. For preparation of lentivirus, 1.0 × 10^6^ HEK-293T cells were transfected with 1.5 μg PAX2 (Addgene, 12260) + 0.3 μg MD2g (Addgene, 12259) + 1.5 μg plasmid in Opti-MEM media (Thermo Fisher Scientific). DMEM + 30% FBS was used to collect virus-containing media at 24 and 48 hours, which was spun down and cleared through a 0.45 μm filter before being added to cells with 0.8 μg/mL polybrene. For *RB1* CRISPR cells, puromycin selection was performed at 1 μg/mL (MDA-MB-231, MCF-7) or 2 μg/mL (T47D, CAL-120), and single clones were isolated and used for all assays. Cas9-only control cell lines utilized an *AAVS1* control guide (5′ CACCGGGGGCCACTAGGGACAGGAT 3′). For *TP53* CRISPR MCF-7 and CAL-51 cells, hygromycin selection was performed at 500 μg/mL.

### Mutagenesis.

The GFP-RB plasmid (Addgene, 16004) was mutated using PCR by introducing 4 synonymous mutations into phosphorylated primers targeting the *RB1* sequence recognized by the gRNA. Q5 polymerase (NEB, M0491) and Taq DNA ligase (NEB, M0647S) were used according to the manufacturer’s protocol (including cycling parameters) for PCR extension and ligation of the plasmid with 2 sequential rounds of mutagenesis (2 bp changes each time). The PCR was DpnI treated (NEB, R0176L) for 1 hour at 37°C and transformed into XL10 Gold bacteria (Agilent, 200314). Colonies were expanded for minipreps (Qiagen, 27104) and Sanger sequenced (U6 primer) to confirm successful mutagenesis.

### Proliferation assays.

Cells plated in 96-well plates were treated with a CDK4/6 inhibitor at concentrations from 0 to 10 μM. After 72 hours, alamarBlue (Thermo Fisher Scientific, DAL1025) was added to the wells at a concentration of 10% of the well volume. After incubation, viability was calculated with the relative absorbance from each well using a plate reader.

### Irradiation.

Irradiation was conducted at the University of Michigan Experimental Irradiation Core using a Kimtron IC-225 at a dose rate of approximately 2 Gy/min. The irradiator was calibrated using dosimetry directly traceable to a National Institute of Standards and Technology (NIST) standard. In vitro experiments were performed with a 0.1 mm added Cu filter and a half-value length of 0.51 mm Cu. In vivo experiments were performed with a Thoraeus filter (0.4 mm Sn + 0.25 mm Cu) and a half-value length of 2.29 mm Cu.

### Immunoprecipitation.

Cells were plated in 10 cm dishes and transfected the next morning with 6 μg GFP-RB plasmid, 6 μg myc-RAD51 plasmid, or the combination. Twenty-four hours later, cells were lysed in 1.0 mL RIPA buffer with protease and phosphatase inhibitor, rocked for 1 hour at 4°C and then spun down, and the pellet was discarded. A BCA assay was used to standardize protein concentrations: approximately 100 mg was removed for input and approximately 1000 mg was used for the IP. Lysates were precleared by incubation with 20 μL of A/G Plus agarose beads (sc-2003) for 1 hour at 4°C; then, GFP-Trap Magnetic Beads (Chromotek, GTD-20), Myc-Trap Magnetic Beads (Chromotek, YTMA-20), or Binding Control Magnetic Agarose Beads (Chromotek, BMAB-20) were added to the cleared lysate, which was left to slowly rock overnight at 4°C. Beads were washed the next morning with RIPA buffer, and bound proteins were eluted in 50 μL RIPA buffer with 1× NuPAGE and 2% β-mercaptoethanol.

### Statistics.

In vitro experiments are graphed as the average ± SEM. Experimental conditions in clonogenic survival assays and HR/NHEJ reporter assays were compared with vehicle (DMSO) controls using a 1-way ANOVA with Dunnett’s post hoc test. Immunofluorescence experiments were blinded for analysis of RAD51 or γH2AX and analyzed with an unpaired, 2-tailed Student’s *t* test between paired RT and combination values for each group, with a correction for the number of time points (6 and 16 hours after radiotherapy). Xenograft tumors were randomized on the first day of treatment, and tumor volume was compared using a 1-way ANOVA at the study end point.

### Study approval.

All xenograft mouse model experiments were done with consent from the IACUC at the University of Michigan.

## Author contributions

Conception and design were contributed by AMP and CWS. Development and methodology were contributed by AMP, TSL, and CWS. Acquisition of data was contributed by AMP, NHH, ARM, KMJ, KWR, ML, LML, CAN, and CW. Analysis and interpretation of data were contributed by ANP, NHH, ARM, KMJ, KWR, BCC, ML, LML, CAN, CW, EFC, TSL, LJP, JMR, and CWS. Writing, review, and/or revision of manuscript were contributed by AMP, NHH, ARM, KMJ, KWR, BCC, ML, LML, CAN, CW, EFC, TSL, LJP, JMR, and CWS. Study supervision was contributed by AMP, TSL, LJP, and CWS.

## Supplementary Material

Supplemental data

## Version 1 12/21/2021

In-Press Preview

## Version 2 02/08/2022

Electronic publication
