# Supplementary material for: RB expression confers sensitivity to CDK4/6 inhibitor–mediated radiosensitization across breast cancer subtypes
Source: JCI Insight. 2022 Feb 8;7(3):e154402. doi: 10.1172/jci.insight.154402 (PMC8855810; doi:10.1172/jci.insight.154402)
Supplement: Supplemental data [file jciinsight-7-154402-s129.pdf]

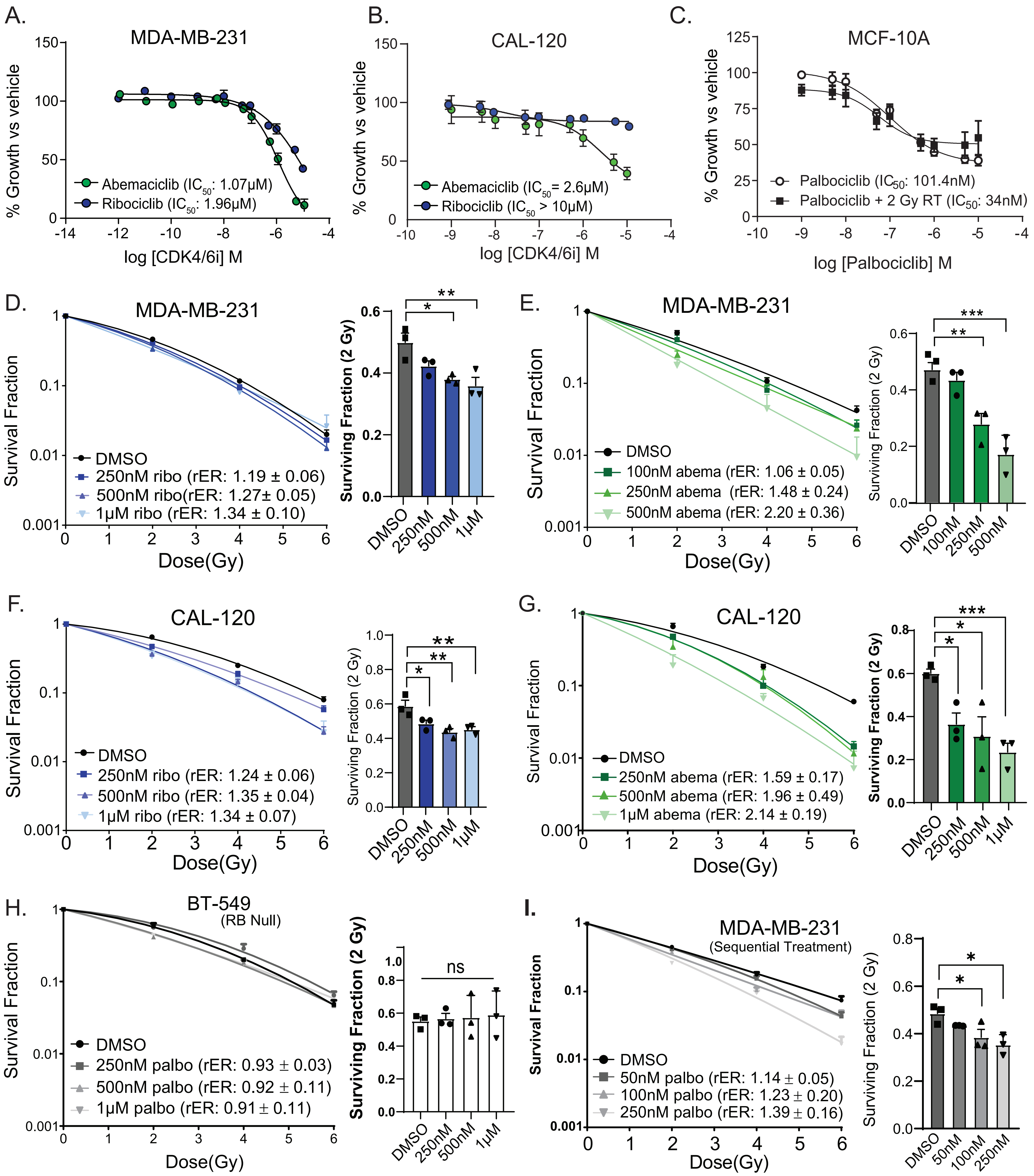

Supplemental Figure S1: Abemaciclib and ribociclib radiosensitize TNBC with wild-type *RB1*

Cell viability was measured in each cell line 72 hours after treatment with either abemaciclib (green) or ribociclib (blue) in RB wild type MDA-MB-231 (**A**) and CAL-120 (**B**) cells to calculate IC_{50} values. MCF10A cells (**C**) were treated with palbociclib alone (open circles) or palbociclib + RT (filled squares). Clonogenic survival assays were performed in MDA-MB-231 (**D-E**) and CAL-120 (**F-G**) cells with varying doses of either ribociclib or abemaciclib and a one-hour drug pretreatment. RB null BT-549 cells were treated with palbociclib + RT (**H**). MDA-MB-231 cells were treated with palbociclib 6 hours after RT, instead of a one hour drug pretreatment (**I**). SF 2 Gy graphs represent the mean of three independent experiments, and for each experiment a one-way ANOVA with Dunnett's post hoc test was used to compare combination-treated groups to the control group treated with RT alone (*, $P < 0.05$; **, $P < 0.01$; ***, $P < 0.001$).

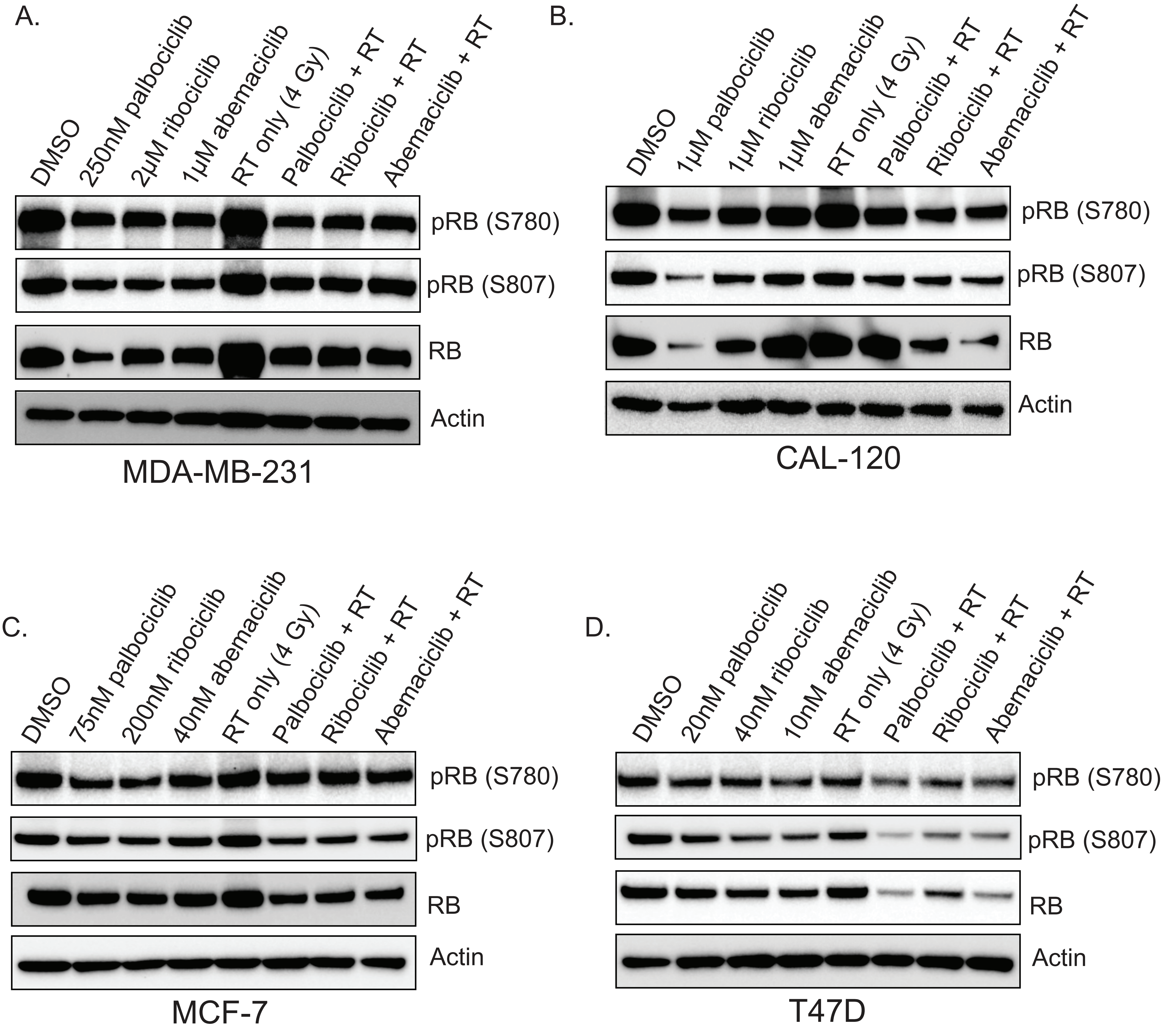

Supplemental Figure S2: **pRB levels decrease with CDK4/6 inhibition**

Western blots were used to assess expression of pRB (S807), pRB (S780), and total RB in RB expressing breast cancer cell lines including MDA-MB-231 (**A**), CAL-120 (**B**), MCF-7 (**C**), and T47D (**D**) cells. Cells were pretreated with a CDK4/6 inhibitor one hour prior to radiation (4 Gy) and harvested 30 minutes after radiation treatment.

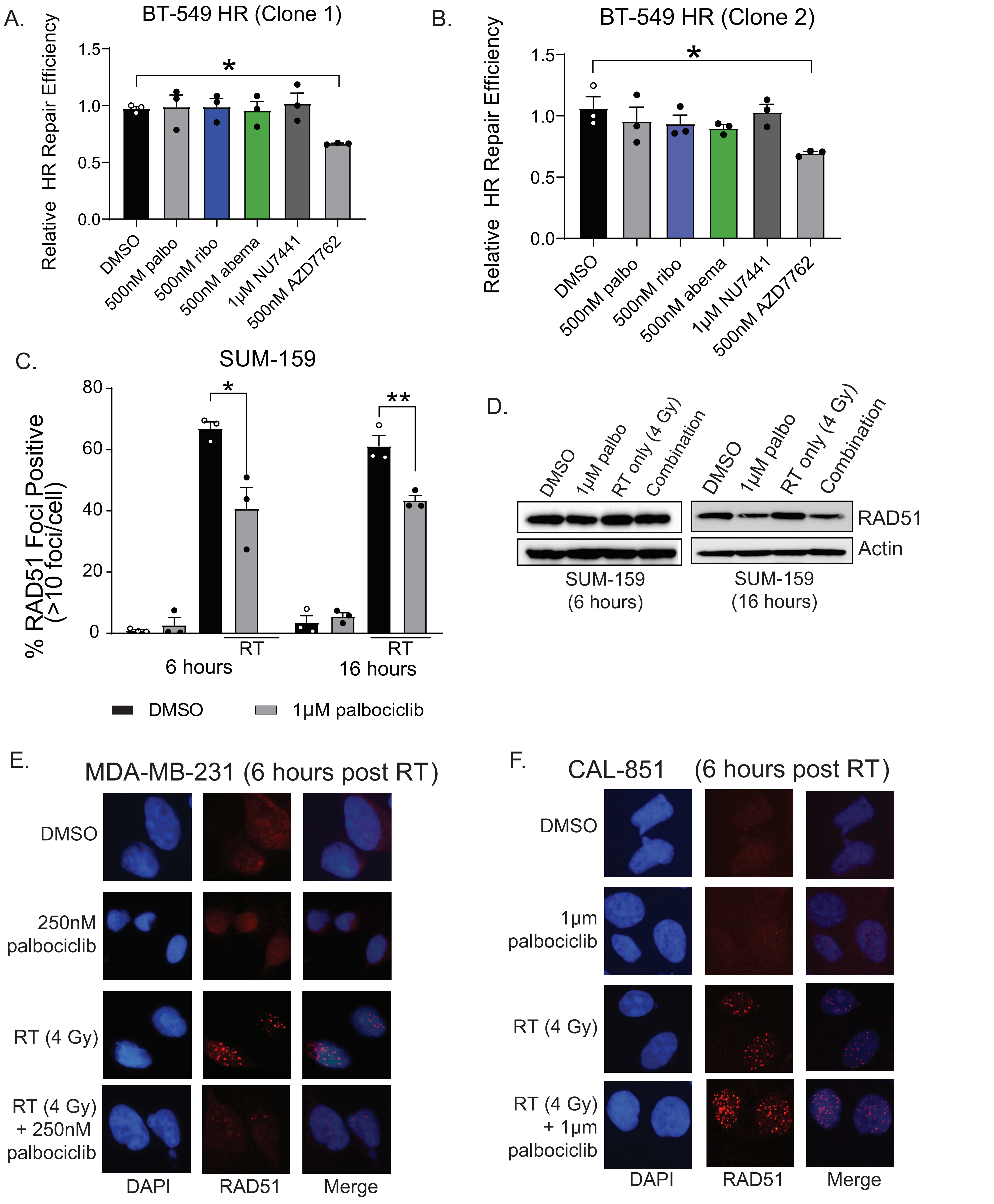

Supplemental Figure S3: **<u>CDK4/6 inhibition impairs HR in TNBC *in vitro*</u>**

Two stable BT-549 HR reporter clones were pretreated ± 500nM CDK4/6 inhibitor one hour before SceI-induction of dsDNA breaks (**A,B**). After a 1-hour pretreatment with ± 1μM palbociclib and ± 4 Gy radiation, coverslips were stained for RAD51 foci 6 hours and 16 hours after radiation in RB wild type SUM-159 cells (**C**). T-tests were performed between paired radiation and combination treated groups at each timepoint, correcting for multiple comparisons. Western blots were used to assess RAD51 protein expression (**D**). Representative images of RAD51 foci (red) 6 hours post radiation are shown in MDA-MB-231 cells (**E**) and CAL-851 cells (**F**).

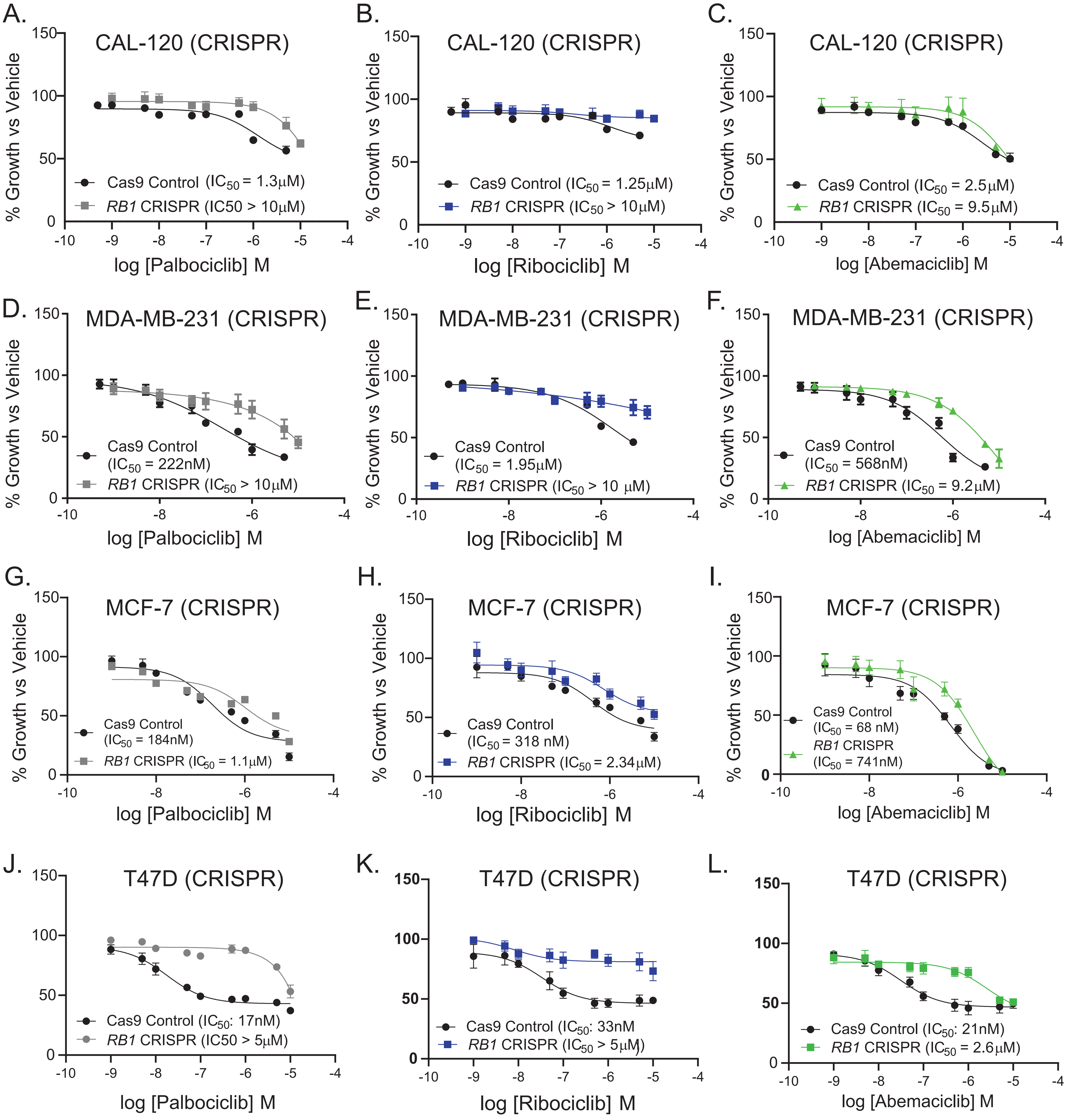

Supplemental Figure S4: <u>*RB1* knockout decreases CDK4/6 inhibitor potency in ER+ and TNBC cell lines</u>

Cell viability was measured 72 hours after treatment with either palbociclib (grey), ribociclib (blue), or abemaciclib (green) in Cas9-expressing control cell lines (black circles) or *RB1* CRISPR knockout cells (colored squares). Dose-response curves were generated for each drug in CAL-120 (**A-C**), MDA-MB-231 (**D-F**), MCF-7 (**G-H**), and T47D (**J-L**) cell lines to calculate IC_{50} values after *RB1* knockout. IC_{50} experiments represent the aggregate of 3 independent replicates and data points display average with SEM.

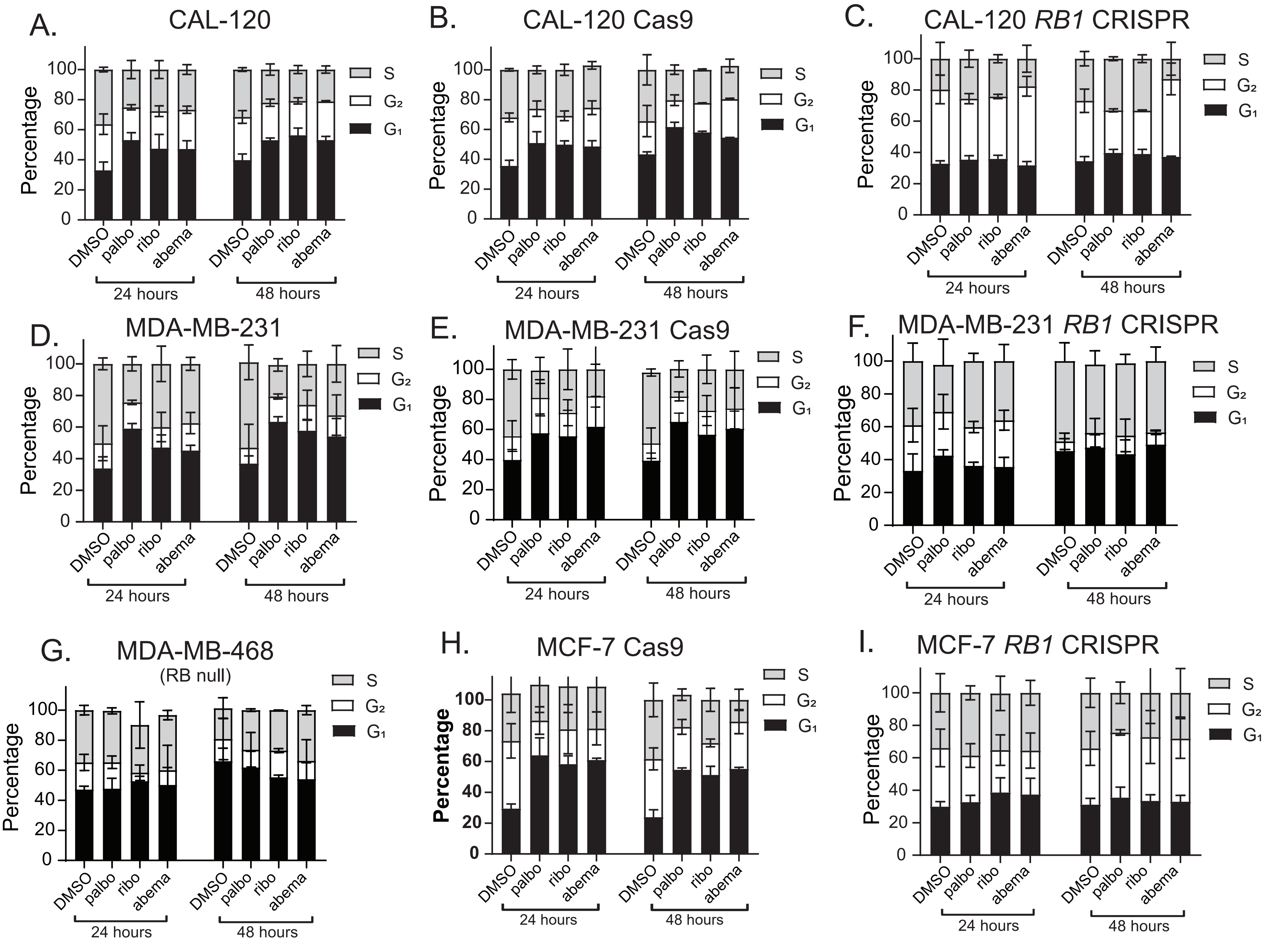

Supplemental Figure S5: *RB1* is essential for G_1 cell-cycle arrest

Flow cytometry was used to quantify cell cycle distribution in G_1 (black), G_2 (white), and S (grey) phase after CDK4/6 inhibition using propidium iodine (PI) staining in CAL-120 cells (**A, B**), MDA-MB-231 cells (**D,E**) and MCF-7 cells **(H)** with intact *RB1*. Cell cycle progression was also quantified in RB null MDA-MB-468 cells (**G**) and in models of *RB1* knockout (**C,F,I**). Cells were fixed at 24 hours and 48 hours post drug treatment with the IC_{50} concentration of palbociclib, abemaciclib, and ribociclib in all cell lines.

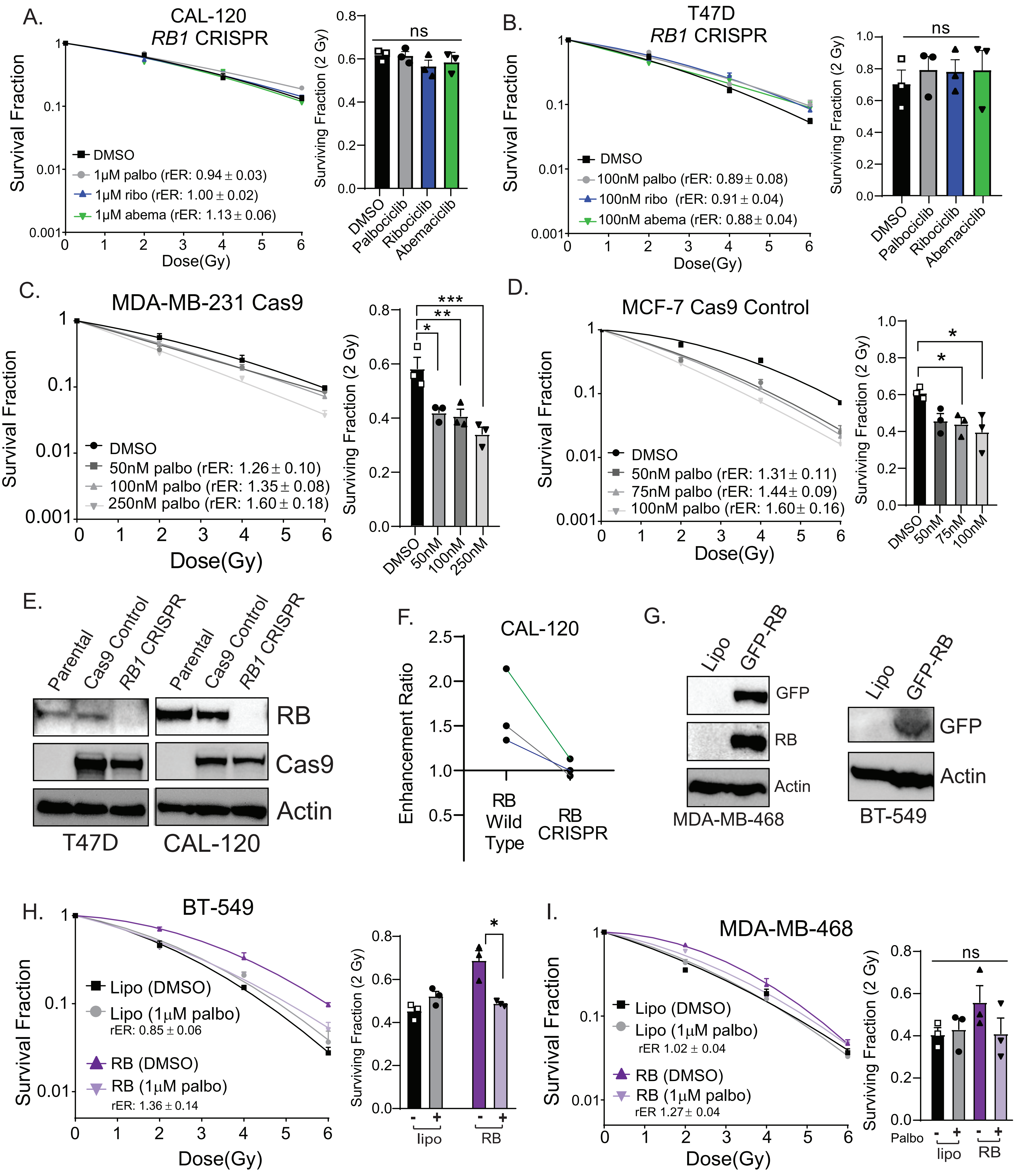

Supplemental Figure S6: **Loss of *RB1* diminishes CDK4/6 inhibitor-mediated radiosensitivity**

Clonogenic survival assays were performed in CAL-120 (**A**) and T47D (**B**) *RB1* CRISPR cells along with MDA-MB-231 (**C**) and MCF-7 (**D**) Cas9 control cells to quantify radiosensitization and calculate radiation enhancement ratios (rER). Western blots were used to confirm successful knockout of RB in CAL-120 and T47D cells (**E**)**.** rER were compared between parental CAL-120 cells and CAL-120 *RB1* CRISPR knockout cells (**F**). Overexpression of RB was performed in MDA-MB-468 and BT-549 cells in order to assess radiosensitivity in clonogenic survival assays (**G-I**). All clonogenics represent the pooled results of 3 independent replicates. (*, $P < 0.05$; **, $P < 0.01$; ***, $P < 0.001$, ****, $P < 0.0001$).

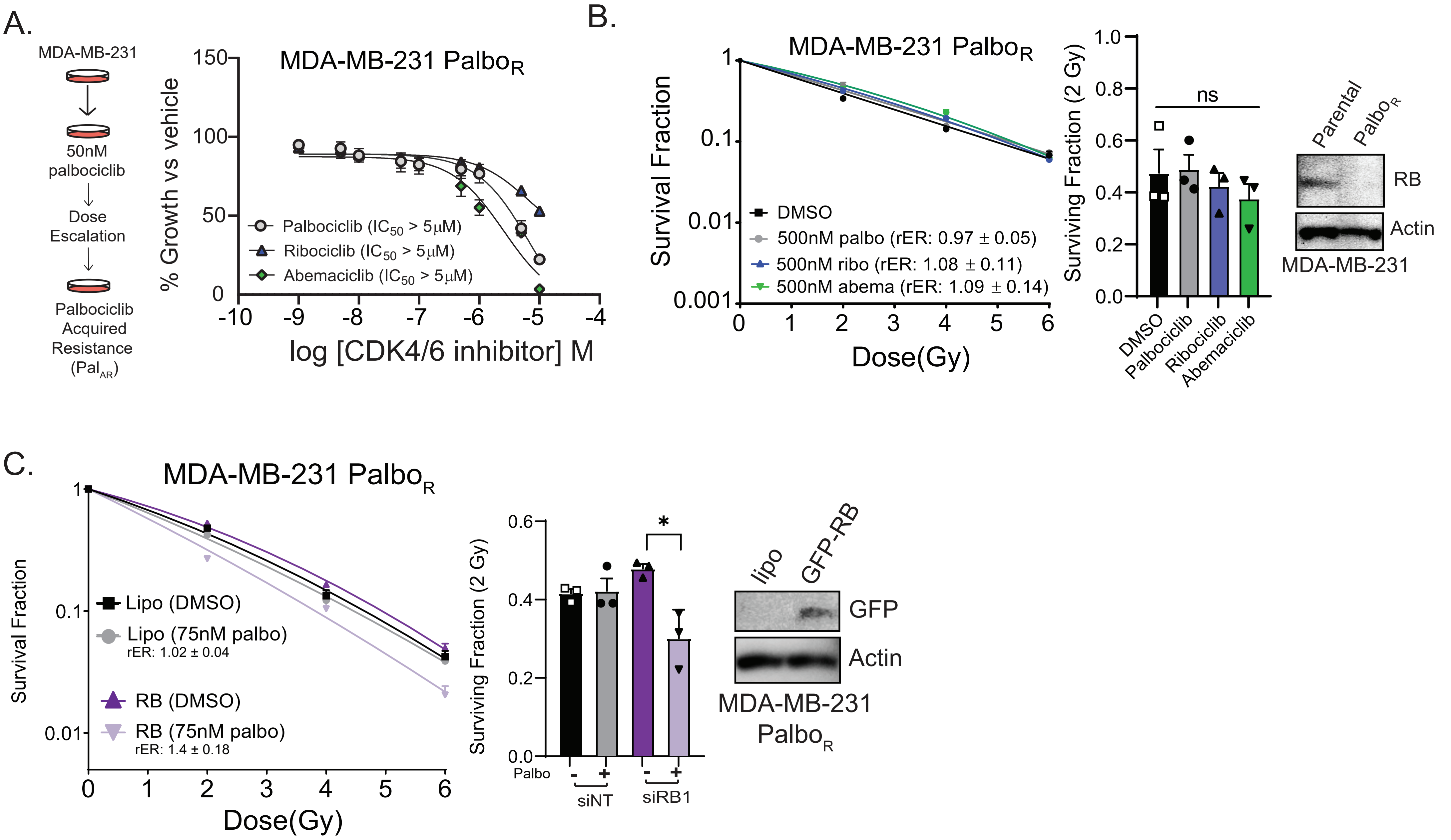

Supplemental Figure S7: **Palbociclib-resistant TNBC cells demonstrate loss of both RB protein and CDK4/6 inhibitor-mediated radiosensitization.**

Palbociclib-resistant MDA-MB-231 cells were generated through continuous culture in drug-containing media (A) and 72 hour cell viability was used to assess acquired resistance to palbociclib, ribociclib, or abemaciclib. Clonogenic survival assays were performed to quantify the rER for PalboR cells and western blots were used to assess RB expression compared to parental MDA-MB-231 cells (B). Radiosensitization was assessed after transient overexpression of GFP-RB and pretreatment ± palbociclib (C).

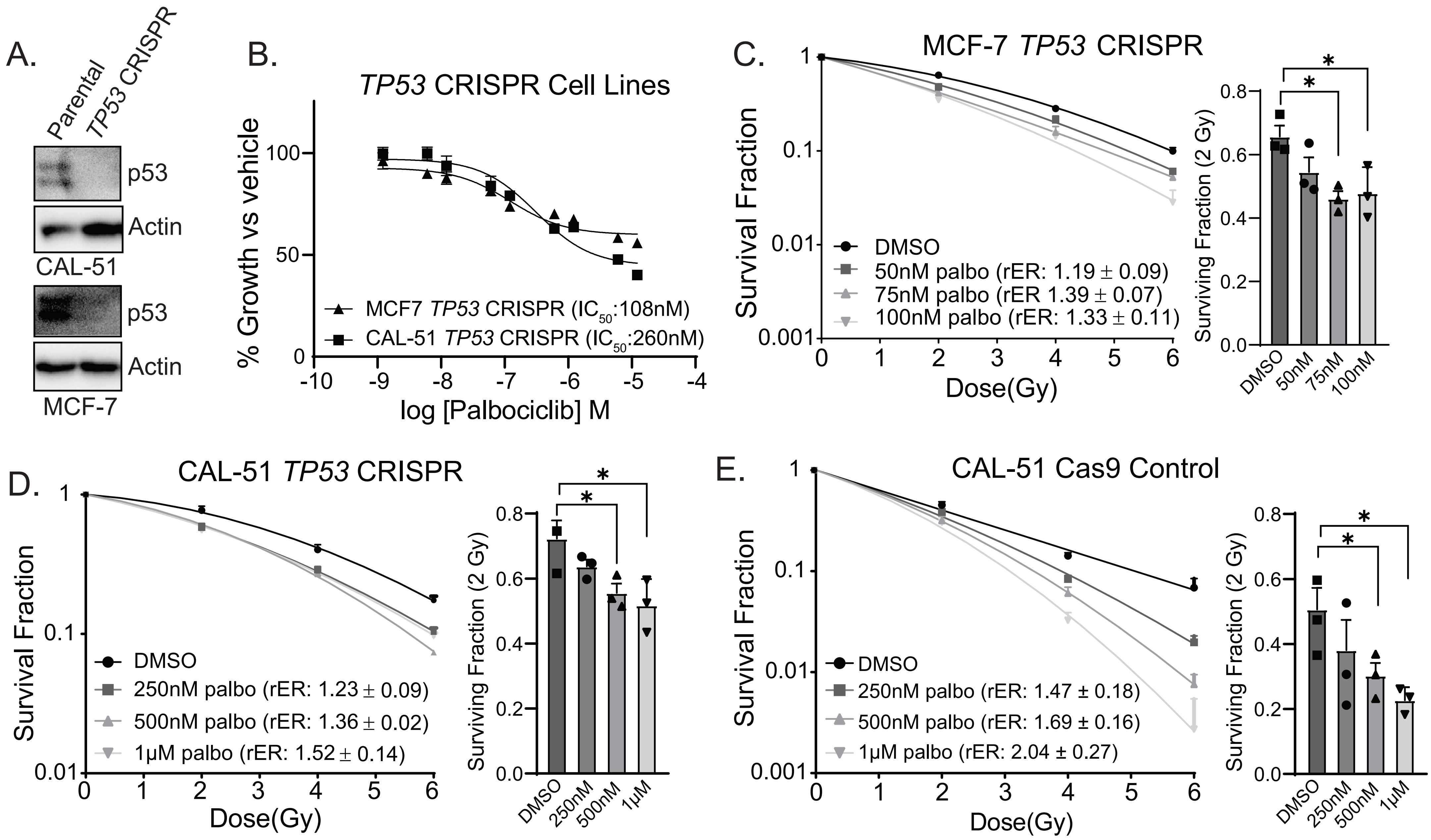

Supplemental Figure S8: **Loss of p53 expression does not significantly impact radiosensitization**

Knockout of p53 protein was assessed by western blot (**A**) and the viability of *TP53* CRISPR cell lines was assessed using Alamar Blue 72 hours after treatment with varying concentrations of palbociclib (**B**). Clonogenic survival assays were used to assess palbociclib-mediated radiosensitization in Cas9 control cells (**E**) and *TP53* knockout cell lines (**C,D**) and using a one hour pretreatment. A one-way ANOVA with Dunnett's post hoc test was used to compare SF 2 Gy values for each cell line (*, $P < 0.05$).

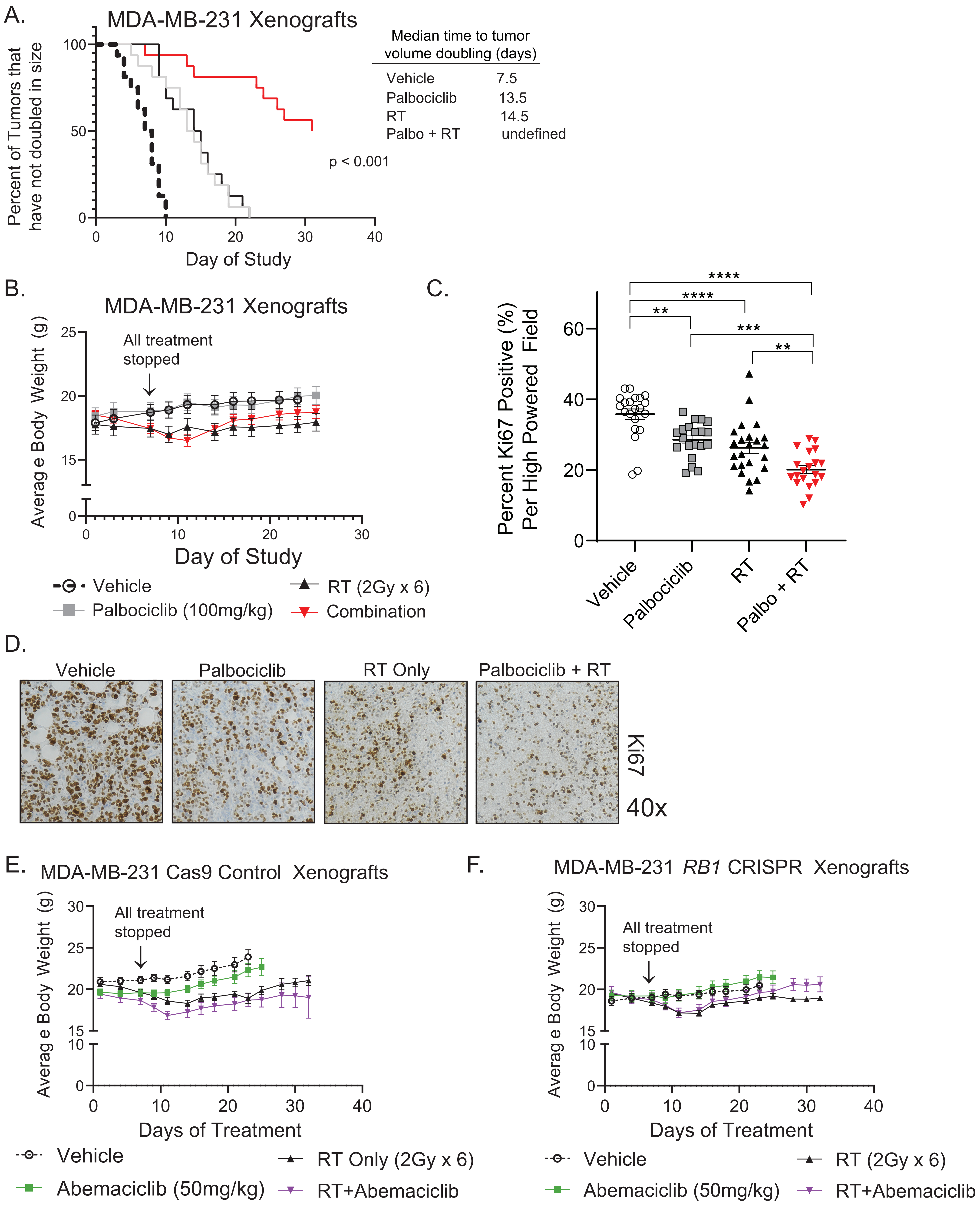

Supplemental Figure S9: <u>CDK4/6 inhibitor-mediated radiosensitization of TNBC *in vivo*</u>

Time to tumor doubling is shown for MDA-MB-231 parental (RB wild type, **A**) xenografts treated with palbociclib, and a log-rank (Mantel-Cox) test was used to compare survival curves. Weights are shown for mice with parental (**B**), Cas9 control (**E**), and *RB1* CRISPR (**F**) xenografts throughout the study. Ki67 staining (imaged at 40x) was used to assess proliferation of tumor cells in mice treated with short term palbociclib and/or RT (**C,D**). A one way ANOVA with Tukey's post hoc test was used to compare Ki67 staining across treatment groups. (*, $P < 0.05$; **, $P < 0.01$; ***, $P < 0.001$, ****, $P < 0.0001$).

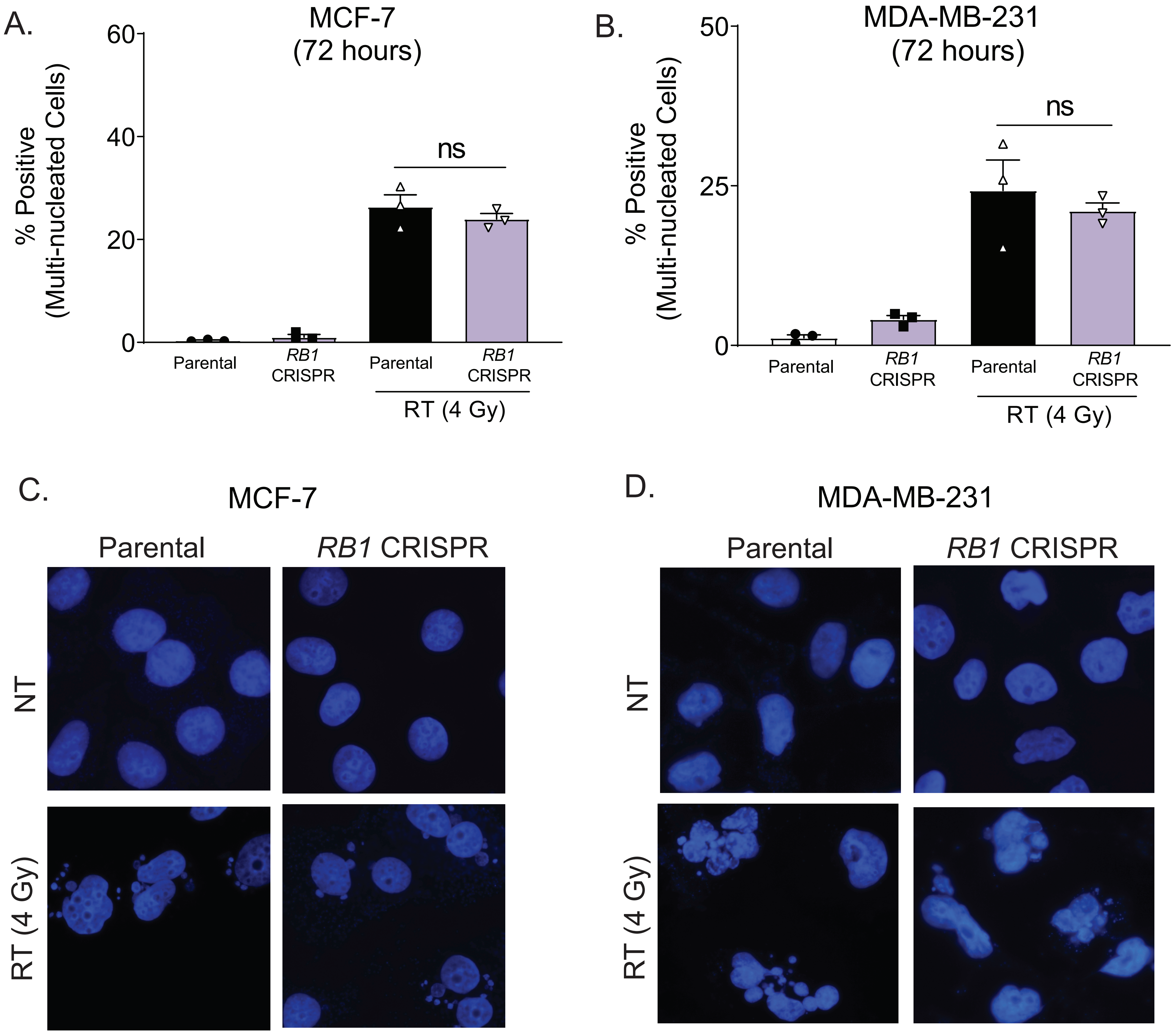

Supplemental Figure S10: **<u>*RB1* loss does not result in increased micronuclei formation following ionizing RT.</u>** Immunofluorescence was used to quantify micronuclei formation following 4 Gy RT in parental and *RB1* CRISPR MCF-7 (**A**) and MDA-MB-231 cells (**B**) 72 hours after RT. Representative DAPI-stained slides are shown for all treatment groups (**C,D**).

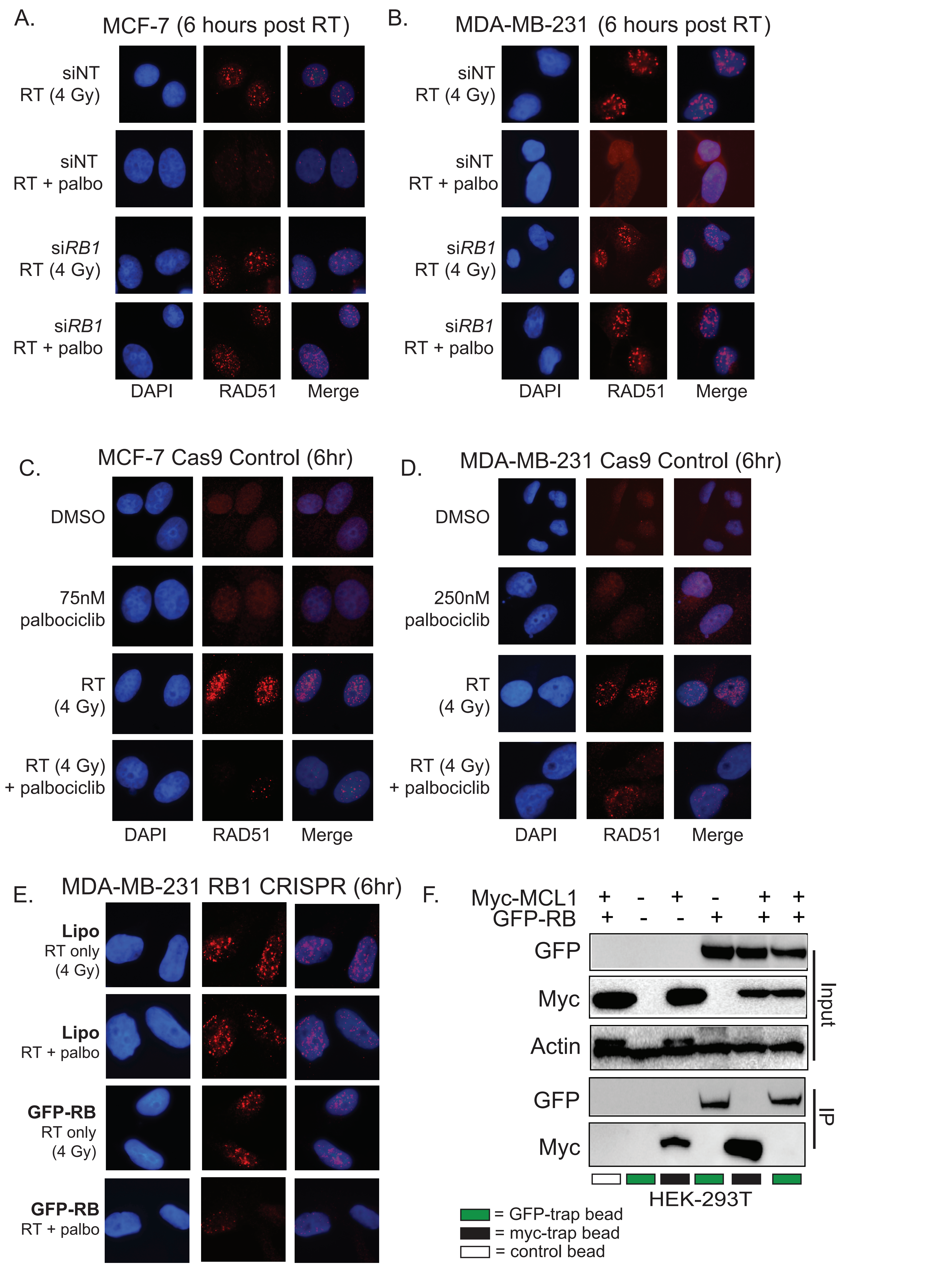

Supplemental Figure S11: **CDK4/6 inhibition suppresses RAD51 foci formation**

Representative images of RAD51 foci at 6 hours post RT (4 Gy) are shown for parental RB wild type MCF-7 (**A**) and MDA-MB-21 (**B**) cells transfected with control or *RB1*-targeting siRNA. Images are also shown for MCF-7 Cas9 (**C**) and MDA-MB-231 Cas9 (**D**) and *RB1* CRISPR (**E**) cells. GFP-RB and myc-MCL1 were transfected into HEK-293T cells and immunoprecipitation was performed 24 hours later **(F).**

	Palbociclib			Ribociclib			Abemaciclib		
	IC_{50}	CRISPR *RB1* IC_{50}	Cas9 Only IC_{50}	IC_{50}	CRISPR *RB1* IC_{50}	Cas9 Only IC_{50}	IC_{50}	CRISPR *RB1* IC_{50}	Cas9 Only IC_{50}
**MDA-MB-231**	241.6nM	>10 µM	222nM	1.99µM	>10 µM	1.95µM	1.07µM	9.2 µM	568nM
**CAL-120**	2.9µM	>10 µM	1.3µM	1.77µM	>10 µM	1.25µM	2.61µM	9.5 µM	2.54µM
**T47D**	20nM	> 5µM	17nM	40nM	> 5 uM	33nM	10nM	2.6µM	21nM
**MCF-7**	75nM	1.047 µM	184nM	200nM	741 nM	317.7nM	40nM	2.34µM	68nM

Supplemental Table S1: IC_{50} values for parental and CRISPR TNBC and ER+ breast cancer cell lines

	Cell Line	IC_{50}	RB
**Palbociclib**	SUM-159	5.5µM	Wild Type
	CAL-51	330nM	Wild Type
	MDA-MB-468	>10µM	Null
	CAL-851	>10µM	Null

Supplemental Table S2: IC_{50} values for additional TNBC cell lines

	Cell Line	Concentration	rER	RB
**Palbociclib**	MCF-7	100nM	1.52 + 0.14	Wild Type
	T47D	25nM	1.50 + 0.13	Wild Type
	CAMA1	75nM	1.32 + 0.09	Wild Type
	ZR-75-1	100nM	1.27 + 0.14	Wild Type

Supplemental Table S3: Radiation enhancement ratios for ER+ breast cancer cell lines treated with palbociclib

	Palbociclib			Combination		
	Day	RT	Palbo	Expected	Observed	Ratio
**MDA-MB-231**	7	0.790	0.762	0.602	0.651	0.925
	16	0.455	0.572	0.260	0.325	0.801
	18	0.381	0.490	0.187	0.257	0.725
	21	0.348	0.476	0.165	0.215	0.768
	Final	0.462	0.866	0.400	0.238	**1.682**

Supplemental Table S4: Fractional Tumor Volume Calculations for MDA-MB-231 xenografts treated with palbociclib.

	Abemaciclib			Combination		
	Day	RT	Abema	Expected	Observed	Ratio
**MDA-MB-231 Cas9 CRISPR**	7	0.738	0.678	0.500	0.624	0.801
	11	0.625	0.608	0.380	0.446	0.852
	16	0.482	0.703	0.338	0.329	1.030
	18	0.448	0.712	0.319	0.299	1.067
	Final	0.549	0.872	0.479	0.320	1.499
**MDA-MB-231 *RB1* CRISPR**	7	0.782	0.818	0.640	0.684	0.935
	11	0.546	0.613	0.335	0.444	0.754
	16	0.440	0.579	0.254	0.327	0.778
	18	0.416	0.591	0.246	0.298	0.824
	Final	0.427	0.721	0.308	0.327	0.939

Supplemental Table S5: Fractional Tumor Volume Calculations for MDA-MB-231 Cas9 Control and *RB1* CRISPR xenografts treated with abemaciclib.

	Timepoint	Treatment	Average Foci / Cell	Average Foci / Positive Cell
**CAL-120**	**6 hours**	*DMSO*	3.66 ± 0.74	
		Palbociclib	2.82 ± 1.34	
		RT (4 Gy)	11.28 ± 3.39	19.56 ± 6.37
		RT + Palbo	7.38 ± 3.02	26.71 ± 10.37
	**16 hours**	*DMSO*	0.91± 0.64	
		Palbociclib	1.14 ± 0.67	
		RT (4 Gy)	11.86 ± 2.01	21.51 ± 2.00
		RT + Palbo	3.48 ± 0.92	17.46 ± 6.07
**MDA-MB-231**	**6 hours**	*DMSO*	6.67 ± 4.77	
		Palbociclib	2.84 ± 1.65	
		RT (4 Gy)	12.81 ± 4.85	25.30 ± 8.16
		RT + Palbo	4.12 ± 2.89	31.92 ± 15.83
	**16 hours**	*DMSO*	1.54 ± 0.49	
		Palbociclib	1.85 ± 0.34	
		RT (4 Gy)	6.43 ± 0.55	12.07 ± 1.01
		RT + Palbo	4.47 ± 3.02	18.11 ± 7.32
**MDA-MB-468**	**6 hours**	*DMSO*	2.72 ± 1.13	
		Palbociclib	2.18 ± 1.96	
		RT (4 Gy)	15.57 ± 4.02	29.13 ± 8.61
		RT + Palbo	11.39 ± 1.62	19.67 ± 3.47
	**16 hours**	*DMSO*	1.92 ± 0.52	
		Palbociclib	1.86 ± 0.84	
		RT (4 Gy)	9.04 ± 0.51	18.18 ± 1.39
		RT + Palbo	7.66 ± 0.32	15.51 ± 1.52
**CAL-851**	**6 hours**	*DMSO*	1.07 ± 0.47	
		Palbociclib	1.27 ± 0.80	
		RT (4 Gy)	16.17 ± 2.71	44.24 ± 10.49
		RT + Palbo	14.51 ± 3.80	34.48 ± 7.25
	**16 hours**	*DMSO*	1.51 ± 0.60	
		Palbociclib	2.46 ± 0.56	
		RT (4 Gy)	7.97 ± 2.33	16.06 ± 4.38
		RT + Palbo	9.32 ± 2.95	20.84 ± 6.95

Supplemental Table 6: Quantification of RAD51 foci in RB wild type and RB null TNBC cell lines.

	Timepoint	Treatment	Average Foci / Cell	Average Foci / Positive Cell
CAL-120	**30 min**	*DMSO*	2.29 ± 0.52	
		Palbociclib	2.31 ± 0.30	
		RT (4 Gy)	20.40 ± 1.03	22.58 ± 0.64
		RT + Palbo	21.16 ± 1.68	24.21 ± 0.89
	**6 hours**	*DMSO*	2.73 ± 1.40	
		Palbociclib	2.51 ± 0.47	
		RT (4 Gy)	17.05 ± 0.60	21.51 ± 0.35
		RT + Palbo	14.51 ± 0.29	17.49 ± 0.54
	**16 hours**	*DMSO*	2.77 ± 1.04	
		Palbociclib	1.16 ± 0.65	
		RT (4 Gy)	13.40 ± 4.23	26.06 ± 0.34
		RT + Palbo	10.85 ± 2.5	24.70 ± 1.42
	**24 hours**	*DMSO*	1.55 ± 0.59	
		Palbociclib	1.87 ± 1.02	
		RT (4 Gy)	7.32 ± 1.11	20.83 ± 4.66
		RT + Palbo	8.27 ± 1.45	20.80 ± 3.96
MDA-MB-231	**30 min**	*DMSO*	1.24 ± 0.51	
		Palbociclib	0.91 ± 0.34	
		RT (4 Gy)	12.84 ± 5.96	19.20 ± 0.90
		RT + Palbo	18.94 ± 1.36	21.34 ± 0.80
	**6 hours**	*DMSO*	2.28 ± 0.76	
		Palbociclib	0.50 ± 0.02	
		RT (4 Gy)	11.63 ± 3.57	14.76 ± 3.05
		RT + Palbo	12.07 ± 2.32	18.76 ± 2.71
	**16 hours**	*DMSO*	1.02 ± 0.13	
		Palbociclib	1.29 ± 0.49	
		RT (4 Gy)	10.08 ± 2.02	23.11 ± 1.52
		RT + Palbo	7.00 ± 4.85	20.52 ± 1.01
	**24 hours**	*DMSO*	0.69 ± 0.18	
		Palbociclib	1.17 ± 0.54	
		RT (4 Gy)	3.78 ± 0.91	18.75 ± 3.49
		RT + Palbo	2.52 ± 1.30	20.17 ± 2.21

Supplemental Table S7: Quantification of γH2AX foci after RT and CDK4/6 inhibition.

Timepoint		Treatment	Average Foci / Cell	Average Foci / Positive Cell
**MCF-7**	**6 hours**	*RT (siNT)*	17.07 ± 0.17	33.04 ± 0.66
		RT + Palbo (siNT)	4.71 ± 1.07	20.71 ± 5.63
		RT (siRB1)	6.5 ± 0.82	17.40 ± 2.98
		RT + Palbo (siRB1)	10.13 ± 1.72	23.74 ± 2.73
**MDA-MB-231**	**6 hours**	*RT (siNT)*	11.57 ± 3.75	28.58 ± 3.25
		RT + Palbo (siNT)	5.65 ± 0.37	18.68 ± 3.01
		RT (siRB1)	5.18 ± 0.38	12.56 ± 0.88
		RT + Palbo (siRB1)	7.99 ± 1.10	21.18 ± 3.10

Supplemental Table S8: Quantification of RAD51 foci in MCF-7 and MDA-MB-231 cells after RT and CDK4/6 inhibition ± si*RB1*.

Treatment			Average Foci / Cell	Average Foci / Positive Cell
**MCF-7 Cas9**	**6 hours**	*NT*	2.51 ± 0.68	
		Palbociclib	1.31 ± 05.1	
		RT (4 Gy)	22.96 ± 4.26	35.79 ± 3.30
		RT + Palbociclib	10.65 ± 1.59	37.61 ± 6.77
**MCF-7 *RB1* CRISPR**	**6 hours**	*NT*	2.02 ± 0.70	
		Palbociclib	0.71 ± 0.29	
		RT (4 Gy)	15.24 ± 2.83	35.38 ± 5.82
		RT + Palbociclib	14.55 ± 3.19	34.82 ± 5.40
**MCF-7 *RB1* CRISPR + GFP-RB**	**6 hours**	*NT*	2.11 ± 1.29	
		Palbociclib	2.42 ± 0.75	
		RT (4 Gy)	19.98 ± 3.42	40.57 ± 7.33
		RT + Palbociclib	6.68 ± 2.87	50.70 ± 12.39
**MDA-MB-231 Cas9**	**6 hours**	*NT*	2.60 ± 1.12	
		Palbociclib	3.61 ± 0.49	
		RT (4 Gy)	24.69 ± 5.25	33.14 ± 5.86
		RT + Palbociclib	8.36 ± 3.24	22.32 ± 9.56
**MDA-MB-231 *RB1* CRISPR**	**6 hours**	*NT*	4.36 ± 1.35	
		Palbociclib	3.29 ± 0.39	
		RT (4 Gy)	12.52 ± 0.76	23.60 ± 0.96
		RT + Palbociclib	9.01 ± 0.23	33.14 ± 2.29
**MDA-MB-231 *RB1* CRISPR + GFP-RB**	**6 hours**	*NT*	1.09 ± 0.73	
		Palbociclib	1.51 ± 0.79	
		RT (4 Gy)	20.42 ± 4.99	45.81 ± 6.48
		RT + Palbociclib	14.97 ± 2.51	38.74 ± 10.26

Supplemental Table S9: Quantification of RAD51 foci in MCF-7 and MDA-MB-231 Cas9 control and RB1 CRISPR cells ± transient GFP-RB overexpression.
